# Optimal Variable Flip Angle Schemes for Hyperpolarized MR Kinetic Modeling Robust to RF Field Variations

**DOI:** 10.1002/nbm.70151

**Published:** 2025-10-11

**Authors:** Marie Frederikke Garnæs, Rie Beck Olin, Pernille R. Jensen, Jan Henrik Ardenkjaer‐Larsen, Kristoffer Hougaard Madsen, Lars G. Hanson

**Affiliations:** ^1^ Department of Health Technology Technical University of Denmark Kongens Lyngby Denmark; ^2^ Department of Applied Mathematics and Computer Science Technical University of Denmark Kongens Lyngby Denmark; ^3^ Danish Research Centre for Magnetic Resonance, Department of Radiology and Nuclear Medicine Copenhagen University Hospital ‐ Amager and Hvidovre Copenhagen Denmark

**Keywords:** hyperpolarized carbon‐13 magnetic resonance, optimal experiment design, pharmacokinetic modeling

## Abstract

Hyperpolarized carbon‐13 magnetic resonance has enabled the real‐time observation of biochemical pathways in living cellular systems. Pharmacokinetic modeling of such experiments provides estimates of conversion rates between metabolites, which, in turn, can be used to distinguish between healthy and diseased tissues, for example. This work focuses on choosing time‐varying flip angle schemes that minimize the uncertainty of model parameter estimates by maximizing the Fisher information while taking into account *B*_1_ field strength variation by incorporating an assumed prior distribution. Monte Carlo simulations demonstrated that the optimized variable flip angle (VFA) schemes provided less uncertain model parameter estimates compared to an optimized constant flip angle (CFA) scheme. Furthermore, the parameter estimation improvement using optimized VFA schemes proved robust over a range of underlying parameters. It was shown that estimation of the *B*_1_ field strength from the measurements is essential to avoid inaccurate parameter estimates from VFA schemes. These may result from violated assumptions about the accuracy of prescribed flip angles. In vitro experiments validated the unidirectional enzyme‐driven conversion model used for demonstration of the optimization methods. Semi‐synthetic data generated through Monte Carlo simulations helped demonstrate the superiority of optimized VFA schemes over an optimized CFA scheme. We conclude that sampling using an optimized VFA scheme and including *B*_1_ as a fitted parameter improves the uncertainties of model parameters as here exemplified by hyperpolarized NMR.

## Graphical abstract

Optimization of variable flip angle schemes that minimize the variances of conversion rate estimates derived from hyperpolarized magnetic resonance experiments. Simulations based on in vitro experiments validated the improved parameter estimation and robustness of the optimized variable flip angle schemes. Additionally, the necessity of incorporating RF field estimation was established.

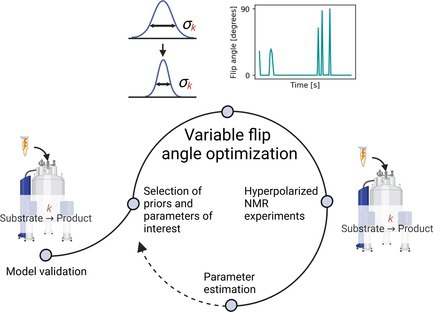

**Abbreviations**
AA: acetoacetate
CFA: constant flip angle
DNP: dynamic nuclear polarization
EAA: ethyl acetoacetate
FIM: Fisher information matrix
L‐BFGS‐B: Limited memory Broyden–Fletcher–Goldfarb–Shanno Bound
MC: Monte Carlo
MRI: magnetic resonance imaging
NMR: nuclear magnetic resonance
PHIP: parahydrogen‐induced polarization
RF: radio frequency
RMSE: root‐mean‐squared error
SEOP: spin‐exchange optical pumping
TR: repetition time
VFA: variable flip angle

## 1 Introduction

Hyperpolarization for nuclear magnetic resonance (NMR) and magnetic resonance imaging (MRI) applications is a powerful tool that has enabled real‐time, pathway‐specific investigation of dynamic metabolic processes in biological systems. It provides nuclear spin polarization much greater than that achieved at normal thermal equilibrium. Hyperpolarization techniques include spin‐exchange optical pumping (SEOP), parahydrogen‐induced polarization (PHIP), and dynamic nuclear polarization (DNP) [1]. Hyperpolarization by dissolution DNP has shown promising results in multiple clinical trials, including several cancer studies [2]. Therefore, DNP is a popular choice for in vivo applications, furthered by its versatility and the commercial availability of polarizers.

Hyperpolarization of a labeled substrate is achieved outside the sample of interest and the substrate is subsequently introduced. The polarization of the substrate and its metabolic products is a limited resource that is lost on the timescale of T_1_ relaxation and additionally reduced by radio frequency (RF) excitation followed by signal readout and decay. While sufficient polarization is available, the substrate and products can be measured time‐resolved, enabling conversion rate estimation. The non‐equilibrium magnetization resulting from hyperpolarization can be modeled using a dynamic hybrid model that combines a pharmacokinetic model and the effects of excitation on the magnetization. The model parameters may include enzymatic conversion rates, which are central parameters that characterize the metabolism in tissue. In the present communication only systems with a single unidirectional conversion rate are considered. Such a rate is typically estimated by fitting parameters to empirical data, for example, for characterizing illness such as cancer [3]. Altered metabolism is a disease indicator and low noise variance of conversion rate estimates may be crucial to determine severity of disease, for example [2, 3, 4]. Other tissue‐specific model parameters of interest may include the T_1_ relaxation rates that depend on the physical and chemical environment [5]. For the needed dynamic measurements, the choice of flip angle sequences affects the metabolite signals and, in turn, the variance of the parameter estimates. Currently used excitation strategies mainly utilize constant low flip angle schemes. This is easy to implement in practice, but schemes using variable flip angles (VFAs) can potentially provide better parameter estimates at limited extra complexity. In addition, shaped RF pulses may be used to excite the metabolites by different flip angles, for example, to conserve substrate [6]. This option is experimentally more challenging and beyond the scope of the current manuscript, although the described methods are also applicable in this case.

In previous work, attempts have been made to find VFA strategies that better estimate enzymatic conversion rates k based on different optimization strategies, such as maximizing the total product signal [7], achieving a constant signal over time [7, 8], or employing T_1_ effective schemes [8]. Although aimed at improving parameter estimates, these prior approaches are not tailored to theoretically do that, which is the focus of the current study. In related studies, Jha et al. [9] and Maidens et al. [10, 11] optimized flip angle schemes to directly minimize the uncertainty of parameter estimates. In the study by Jha et al. mutual information between data and model parameters was utilized, while Maidens et al. used optimal control showing very promising results.

The RF strength of the *B*_1_ field can be challenging to estimate accurately in practice, particularly in vivo. In experiments, the *B*_1_ field strength can be subject to inaccurate RF transmit calibration and can vary across the subject due to imperfect RF transmit coils [12, 13]. The *B*_1_ strength directly affects the flip angles [11], which can affect parameter estimates in the subsequent analysis [12, 14]. Although *B*_1_ maps can be estimated and used for calibration, this approach requires additional steps and does not resolve issues of *B*_1_ inhomogeneity [15, 16].

It is well known that RF miscalibration and *B*_1_ inhomogeneity may compromise and even eliminate the benefits of VFA schemes [17]. This work aims to develop a robust method for determining VFA schemes that estimates an enzymatic conversion rate k with minimal variance over a range of *B*_1_ field strengths. Our approach is based on the work by Maidens et al. [10], in which a scalar‐valued function of the Fisher information matrix (FIM) is used as the objective function to minimize the uncertainties of model parameter estimates. The methods are kept flexible, so that any combination of the model parameters can be prioritized. The effects of prioritizing the variances of all rate parameters are investigated with special focus on the enzymatic conversion rate. *B*_1_ robustness is incorporated into the objective function by replacing the assumption of accurately known *B*_1_, corresponding to a Dirac delta function prior, with a more realistic prior distribution that reflects flip angle uncertainty. Thus, it is ensured that the optimized schemes are effective over a range of *B*_1_ values. Furthermore, *B*_1_ field strength is included as a parameter to be fitted directly, and by doing so, the remaining model parameters are estimated correctly. Monte Carlo (MC) methods are used to demonstrate the robustness of the optimized schemes to variation of the underlying parameters including *B*_1_.

Finally, the application of the mathematical framework is demonstrated through in vitro NMR experiments. The reliability of conversion rate estimation is investigated by repeated experiments using both VFA and constant flip angle (CFA) schemes. It is practically infeasible to directly verify improved parameter estimates, since such a brute‐force approach requires a large number of repeated measurements. Furthermore, it is not necessary, since high‐SNR data can be acquired, and the noise sensitivity can be explored by evaluating simulated low‐SNR data for a number of noise realizations. Hence, a range of high‐SNR NMR experiments with varying underlying parameters was carried out. To obtain estimates of parameter variances, a MC approach was used where semi‐synthetic NMR data were generated to simulate experiment repetition.

## 2 Theory

The theory used in the following has broad applicability and is not limited to a particular model. However, the theory is exemplified for a simple unidirectional metabolic conversion model that is relevant for a number of biological systems, including the conversion of pyruvate to lactate [2] and ethyl acetoacetate (EAA) to acetoacetate (AA) [18], the latter of which will be explored experimentally in this work.

### 2.1 Hyperpolarized NMR Model

The dynamics of the magnetization between excitations in a hyperpolarized experiment can be described using a pharmacokinetic model. To this end, a two‐dimensional system of ordinary differential equations is used to model the kinetics of the unidirectional enzyme‐driven conversion that takes place along with metabolite spin–lattice relaxation:

(1) $$\frac{dx\left(t\right)}{\mathrm{dt}}=\left[\begin{array}{cc} -k-R_{1\;S} & 0\\ k & R_{1\;P} \end{array}\right]x\left(t\right)$$

Here x is a vector containing the state of longitudinal magnetization of substrate and product in its first and second element, respectively. k is the conversion rate from substrate S to product P. $R_{1\;S}=1/T_{1\;S}$ and $R_{1\;P}=1/T_{1\;P}$ are the relaxation rates of the two. At time t, an excitation is made with a selectable flip angle $\alpha_{t}$, and the metabolite signals are subsequently acquired while the transversal magnetization decays to 0 and is furthermore spoiled. The considered experiment uses a broadband pulse, which excites both metabolites simultaneously by the same flip angle. The initial magnetization available before the first excitation at $t_{0}$ is modeled by two parameters $S_{0}$ and $P_{0}$. Right after each of the excitation pulses at time t, transversal magnetization $y_{t}$ of magnitude $\sin\left(\alpha_{t}\right)\cdot x_{t}$ is generated, where $x_{t}$ is the longitudinal magnetization before excitation, after which $\cos\left(\alpha_{t}\right)\cdot x_{t}$ remains. Thus, the evolution of magnetization is described by the pharmacokinetic model between pulses, and the effects of excitation pulses are described by discrete jumps in the system state. The pulses are assumed so short that conversion and relaxation during these are negligible. Therefore, this system is considered a hybrid dynamical system [19]. However, as we are only interested in the system state at the acquisition times, the ordinary differential equation model is discretized in time simplifying it to a discrete‐time dynamical system. Samples are acquired at time points separated by the repetition time TR and the between‐pulse magnetization evolution is described by a transition matrix, G [20]:

(2) $$G\left(\theta \right)=\exp\left(\mathrm{TR}\cdot \left[\begin{array}{cc} -k-R_{1\;S} & 0\\ k & -R_{1\;P} \end{array}\right]\right)$$

corresponding to the discretization of the dynamics in Equation (1). Here θ is a vector of model parameters, including relaxation and conversion rates. Measures proportional to the metabolite concentrations are calculated from the free induction decays following each excitation, and these constitute the elements of the signal vector y. The data D is modeled as a normally distributed random variable leading to the discrete‐time model:
(3)

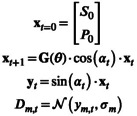

where t+1 refers to the time at one TR after t and the index m labels the metabolic compounds. The model parameters to be fitted are

(4) $$\theta =\left[k,R_{1S},R_{1P},S_{0},P_{0}\right]$$

and the parameters to be chosen in the optimization are the N flip angles

(5) $$\alpha =\left[\alpha_{0},\alpha_{1},\ldots ,\alpha_{N}\right]$$

in order to obtain the best possible estimates of the model parameters θ.

#### 2.1.1 Introducing the RF Field Strength Parameter $B_{1}^{S}$

$B_{1}^{S}$ is introduced as a scaling of the flip angles α to be able to model the difference between the expected and actual flip angles experienced by the metabolites. As a result, the parameter is implemented in the model by updating $x_{t+1}$ and $y_{t}$ in Equations (3) and (4):
(6)

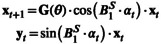

(7) $$\theta =\left[k,R_{1S},R_{1P},S_{0},P_{0},B_{1}^{S}\right]$$

Note that when using the CFA scheme with this version of the model, there is a complete correlation between estimates of the $B_{1}^{S}$ parameter and the parameters $S_{0}$, $P_{0}$, $R_{1S}$, and $R_{1P}$, respectively, meaning they cannot reliably be estimated simultaneously. Thus, when using the CFA scheme, the limited model described in Equation (3) is used, but potentially inaccurate flip angle assumptions will result in biased estimates.

In the remainder of this work, the version of the model described in Equation (3) will be referred to as the model without $B_{1}^{S}$, while the model presented in Equation (6) will be referred to as the model with $B_{1}^{S}$.

### 2.2 Optimal Experiment Design

The aim of optimal experiment design with regard to parameter estimation is to find acquisition parameters that optimize a scalar quality measure based on parameter variance. In model‐based optimal design, the optimal set of measurement conditions is independent of the noise amplitude when the underlying model is linear and the measurement noise is additive, independent, and possessing a constant variance. These characteristics are sufficiently fulfilled for many MR‐relevant situations and are often assumed (implicitly) when model fitting is employed to extract sample parameters. Common optimization criteria include scalar‐valued functions of the FIM as it contains information about the influence of parameters on the output. Such criteria are often feasible to implement [21] and the FIM ℐ can be calculated by [22]:
(8)

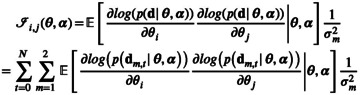

where p is the probability distribution of the data d, indices i and j number the target parameters, and N is the number of data acquisitions. Both the noise standard deviation $\sigma_{m}$ and the trajectories x are subject to normalization by $S_{0}\cdot \sin\left(\alpha_{0}\right)$ to match the normalization of the signal y that takes place before the analysis of the experimental data.

In turn, ℐ can be used to estimate the lower bound of the parameter variance–covariance matrix through the Cramér–Rao inequality [21]:

(9) $$\mathrm{Cov}\left(\hat{\theta }\right)\geq ℐ{\left(\theta ,\alpha \right)}^{-1}$$

To obtain an objective function that can be optimized, some scalar‐valued function of ℐ must be chosen. In this work L‐optimality is utilized:

(10) $$f_{L}\left(ℐ\right)=\mathrm{tr}\left(W\cdot ℐ{\left(\theta ,\alpha \right)}^{-1}\right)$$

The trace of any matrix X is here denoted tr(X). W is a diagonal matrix containing scalar weights that assign importance to individual parameter variances in the diagonal of $\mathrm{Cov}\left(\hat{\theta }\right)$. More commonly known than L‐optimality is A‐optimality, which is the special case of L‐optimality where W=I is the identity matrix [23]. Note that the objective function optimum dictating the optimal flip angle scheme is not affected by the chosen noise level. The noise factor is merely a scaling that does not depend on input parameters meaning the parameter estimate uncertainties scale linearly with the noise, when thermal noise dominates the ever‐present model imperfections.

To ensure a scheme that efficiently fits model parameters over a range of $B_{1}^{S}$ values, a prior was applied to the L‐optimality function. As a result, determination of the optimal flip angle scheme is achieved by optimizing the objective function:

(11) $$\min_{\alpha }\;\int_{\;B_{1}^{S}}f_{L}\left(ℐ\left(\theta ,\alpha \cdot B_{1}^{S}\right)\right)p_{0}\left(B_{1}^{S}\right)\;{\mathrm{dB}}_{1}^{S}$$

Optimizing for flip angles α requires a choice of expected model parameter values θ in advance. These parameter values are referred to as *nominal values* and express prior expectations of the parameters that measurements are performed to determine. In turn, an experiment performed using the optimized scheme will be most sensitive to deviations from the specific chosen nominal values. Such optimized sensitivity is valuable, as the determination of changes in metabolic parameters with known unperturbed values is often the main objective in experiments aiming to characterize, for example, disease or an intervention such as a treatment. Theoretically, a prior distribution can be implemented to incorporate uncertainty about nominal parameters to obtain sensitivity over a range similar to what is performed for $B_{1}^{S}$. This will be elaborated further in Section 5.2.

#### 2.2.1 Computation of FIM ℐ

Since noise is assumed normally distributed in this work, elements in the FIM can be computed with

(12) $${ℐ}_{i,j}\left(\theta ,\alpha \right)=\sum_{t=0}^{N}\sum_{m=1}^{2}\frac{1}{\sigma_{m}^{2}}\cdot \frac{\partial y_{m,t}\left(\theta ,\alpha \right)}{\partial \theta_{i}}\cdot \frac{\partial y_{m,t}\left(\theta ,\alpha \right)}{\partial \theta_{j}}$$

Thus, we need to compute sensitivities as follows:

(13) $$\frac{\partial y_{m,t}\left(\theta ,\alpha \right)}{\partial \theta_{i}}=\sin\left(\alpha_{t}\right)\cdot \frac{\partial x_{m,t}\left(\theta ,\alpha \right)}{\partial \theta_{i}}$$

From Equation (3), they are computed using the product rule:
(14)

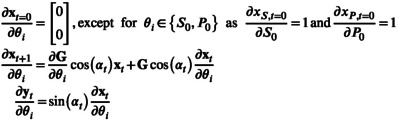




#### 2.2.2 Regularization

As will become evident in Section 4, the optimized VFA schemes tend to exhibit a large number of 0° flip angles and a few large flip angles early in the sequence when significant magnetization is still present. In contrast, pulses following excitations with large flip angles appearing later in the sequence provide very little additional information as the magnetization is essentially depleted. Thus, for these late excitations there is practically no gradient in the objective function to change them away from the initialization, which in this case is random. This is addressed by the introduction of a regularization term which weakly promotes sparsity of the flip angles. Thus, insignificant excitation pulses are avoided and comparison of excitation schemes across multistart optimization is facilitated.

(15) $$n\sigma^{2}\lambda \sum_{t=0}^{N}\sin{\left(\alpha_{t}\right)}^{2}$$

λ denotes a scalar parameter trading off L‐optimality against the penalty. The combined scaling facilitates choosing a suitable λ value across parameter weights W and noise variance $\sigma^{2}$ chosen as $\sigma_{m}^{2}$ for any of the metabolites. n denotes the number of parameters that have been assigned a weight through W. The regularization term implements a squared Euclidean distance metric (L2‐norm), which is equivalent to imposing a Gaussian shrinkage prior on the flip angles and performing a posteriori maximum estimation. The sine that originates in the signal equation makes the bias of large flip angles insignificant and makes the order of magnitude of the sum 1 for near‐optimal schemes that necessarily have few large flip angles as discussed later. Here we set λ=0.1, which, with the chosen normalization of the L‐optimality metric, ensures that the prior is weakly informative. In practice, this causes the regularization to have no effect on early excitations where significant magnetization is present and therefore the L‐optimality metric dominates. During later parts of the schemes, most magnetization has been depleted, causing the convergence to few large non‐zero excitations due to the effect of regularization.

#### 2.2.3 Non‐Dimensionalization

Prior to optimization, the parameters are scaled according to the principles of non‐dimensionalization. This is used to remove physical dimensions from an equation with physical quantities when the variable dynamics are of interest [24]. In the problem at hand, non‐dimensionalization is used to remove units from the equations to simplify the interpretation of weights. Therefore, the obvious choice of non‐dimensionalization constants (units) are $T_{0}=\mathrm{TR}$, and the measured transversal magnetization of the substrate at t=0.

## 3 Methods

The investigation is structured in two distinct phases. The first phase is an in silico analysis, where MC simulations are employed to evaluate optimized flip angle schemes. The second phase involves in vitro experiments, where the optimized flip angle schemes are directly tested using hyperpolarized NMR experiments. The code utilized for optimization and MC simulations in the first phase is available at https://github.com/MarieGarnaes/Optimal‐VFA‐Schemes.

### 3.1 In Silico Optimization

A total of six different flip angle schemes were determined through optimization; one CFA scheme and five VFA schemes deviating by the nominal parameters and weights applied through the diagonal matrix W in Equation (10). Specifics for each of the schemes are listed in Table 1. The weights for $S_{0}$ and $P_{0}$ were kept at 0 for all schemes since the initial magnetizations are only indirectly interesting as indicators of whether other quantities can be measured. Across the measurement schemes, different choices of weights were used for the rates k, $R_{1P}$, and $R_{1L}$. The scheme “CFA‐k” was restricted to CFAs and was specifically weighted for optimal k rate estimation as indicated in the name. Flip angles were allowed to vary over time for the schemes “VFA‐k,” “VFA‐$R_{1S}$,” and “VFA‐$R_{1P}$” that were specifically weighted to optimally estimate each of the corresponding rates. The scheme “VFA‐all” was optimized using weights on *all* rates scaled inversely by nominal parameter size. The scaling was included to allow larger parameters to have a correspondingly larger variance. Lastly, “VFA‐suboptimal” is a scheme optimized for a set of rates that were each 1.5 times larger than the nominal parameters. The purpose was to investigate parameter estimation from data acquired using a scheme that is *suboptimal* for those same parameters, as it was optimized for an inaccurate set of values.

**TABLE 1:** Nominal model parameters and weights selected for various optimized flip angle schemes.

Scheme name	Parameter (s^−1^)			Weights		
	K	$R_{1S}$	$R_{1P}$	k	$R_{1S}$	$R_{1P}$
CFA‐*k*	0.0135	$\frac{1}{35}$	$\frac{1}{54}$	$\frac{1}{k^{2}}$	0	0
VFA‐*k*						
VFA‐$R_{1S}$				0	$\frac{1}{R_{1S}^{2}}$	
VFA‐$R_{1P}$				0	0	$\frac{1}{R_{1P}^{2}}$
VFA‐all				$\frac{1}{k^{2}}$	$\frac{1}{R_{1S}^{2}}$	$\frac{1}{R_{1P}^{2}}$
VFA‐suboptimal	0.02025	$\frac{1.5}{35}$	$\frac{1.5}{54}$	$\frac{1}{k^{2}}$	0	0

The prior was implemented by summation over 11 $B_{1}^{S}$ values according to a distribution of $N\left(1,0.15\right)$, equidistantly spaced over the interval of 0.5–1.5.

The optimization was implemented in Python 3.11.5 with the *scipy.optimize.minimize* function, using the *L‐BFGS‐B* (Limited memory Broyden–Fletcher–Goldfarb–Shanno Bound) minimization method to find the local minima of the objective function. The optimizer takes several input parameters including the tolerance level set to $10^{-9}$ adjusted for parameter and weight sizes. The optional parameters—maximum number of iterations, step size, maximum number of line search steps, and maximum number of function evaluations—were chosen as 10,000, 0.0001, 100, and 100,000, respectively. Bounds were applied to restrict the flip angles to the range from 0° to 90°, except for CFA and $t_{0}$ in VFA for which a lower bound of 0.1° was chosen for numerical reasons. The algorithm requires an initialization scheme that may affect the outcome of the optimization.

#### 3.1.1 Choice of Nominal Parameters

Before the in silico investigation, in vitro NMR data were acquired and the corresponding parameter estimates provided kinetic model validation and a basis for nominal parameter choices. Specifically, enzymatic conversion of EAA to AA was studied. The relaxation time constants $T_{1S}$ and $T_{1P}$ had previously been found to be 35 and 54 s for EAA and AA, respectively. The nominal values of $S_{0}$ and $P_{0}$ were fixed at 5 and 0.1, respectively, for all schemes. This expresses that the substrate concentration is initially high compared to the product concentration. In the NMR experimental design, the enzyme concentration was selected to result in a conversion rate near $k=0.0135\;s^{-1}$, similar to the conversion rate for cancer cells in the work by Jensen et al. [18].

#### 3.1.2 Multistart of Optimization

Since it is unknown if the objective function has multiple local minima, the initialization may influence which minimum is determined if multiple minima exist. Therefore, the optimizations were run using a set of different initializations to increase the chance of determining the global minimum. When optimizing CFA‐k, the CFAs 10°, 20°, 30°, 40°, 50°, 60°, 70°, and 80° were used for initialization. When optimizing the VFA schemes, 25 different initializations were generated by sampling random numbers between 10° and 80° from a uniform distribution.

### 3.2 Validation Using MC Simulations

To validate the benefits of the newly found VFA schemes, a number of MC measurement simulations were performed. Computer‐simulated data collection has advantages over empirical data acquisition, including access to the ground truth parameters used for data generation and the ability to provide a large number of statistically independent datasets. Thus, the parameter estimation error resulting from measurement noise can be estimated, and the efficacy of the different flip angle schemes can be demonstrated. In all of the following MC analyses, the parameters listed in Equation (4) (k, $R_{1P}$, $R_{1L}$, $P_{0}$, and $L_{0}$) will be fitted. In a supplementary analysis, $B_{1}^{S}$ will additionally be included as a model parameter, according to the model used (Equation (7)).

#### 3.2.1 Scheme‐Dependent Distribution of Parameter Estimates

This MC analysis was performed to investigate the distribution of parameters resulting from utilizing the six different flip angle schemes. One thousand simulated datasets were generated by adding different realizations of Gaussian noise to the substrate and product trajectories for each flip angle scheme. The trajectories were generated using the kinetic model from Equation (3) (without $B_{1}^{S}$) and the nominal parameter values described in Section 3.1.1. The chosen noise distribution $N\left(0,5\cdot 10^{-3}\right)$ resulted in ∼2% relative standard deviation of the *k* parameter estimate. The model that was also used for trajectory generation, was fitted to each of the realizations of the simulated data and the resulting parameter estimates were evaluated.

#### 3.2.2 Robustness to Parameter Variation

To examine how well the optimized flip angle schemes perform if the ground truth parameters vary from the nominal values on which the optimization was based, an additional analysis using MC simulations was performed. Trajectories were generated varying one parameter at a time with ±25% and ±50% of the nominal value, while the remaining parameters were fixed to their nominal values. For each of these trajectories, 1000 different noise realizations ($N\left(0,5\cdot 10^{-3}\right)$) were performed to simulate measurements that were subsequently fitted with the model without $B_{1}^{S}$, and the corresponding parameter estimates were evaluated. The same 1000 realizations of noise were used across schemes, parameters and parameter deviations to ease the comparison.

#### 3.2.3 $B_{1}^{S}$ Fitting and Robustness to $B_{1}^{S}$ Variation

In the previous analyses, $B_{1}^{S}$ was assumed known and non‐varying. These assumptions are not made in this MC analysis. Thus, it was examined how the inclusion of $B_{1}^{S}$ as a model parameter affects the remaining parameter estimates and how varying $B_{1}^{S}$ affects the fits. Firstly, 1000 simulated datasets were generated by adding different realizations of noise ($N\left(0,5\cdot 10^{-3}\right)$) to the trajectories for each flip angle scheme. Next, 1000 datasets were generated, each with variation of both the $B_{1}^{S}$ drawn from a Gaussian distribution $N\left(1,0.15\right)$ and the realizations of noise ($N\left(0,5\cdot 10^{-3}\right)$) added. Again, the same 1000 realizations of noise were used across schemes to ease the comparison. The standard deviation of $B_{1}^{S}$ was chosen to realistically reflect the variation found in MRI experiments [16]. Each of these trajectories were subsequently fitted with both models, and the corresponding parameter estimates were evaluated.

### 3.3 In Vitro Experiments

To demonstrate the usefulness of the mathematically derived model and flip angle optimizations, a biologically relevant model system that can be varied and investigated experimentally is needed.

Hepatocellular carcinoma is the most common form of primary liver cancer but is difficult to diagnose by traditional imaging as it often presents in tissue already affected by other underlying diseases [25]. Jensen et al. [18] showed in animal models that a decrease in the activity of the carboxyl esterase enzyme is a marker of hepatocellular carcinoma cancer. This enzyme catalyzes the conversion from EAA to AA. Thus, by injecting the hyperpolarized substrate [1,3‐^13^C] EAA and subsequently measuring it and its product [1,3‐^13^C] AA dynamically, uptake and conversion can be quantified in vivo by MRI. The enzymatic activity can also be measured in vitro by NMR, which simplifies the system to be modeled.

Experiments that can generate high‐SNR data are needed here for model validation, parameter estimation, and to provide the basis for semi‐synthetic data generation used in MC analyses with realistic model deviations. Motivated by this, a small number of high‐SNR experiments were conducted over the course of 2 days:

- Day 1: primary dataset. CFA and VFA schemes, chosen loosely based on prior expectations and optimization, were used in two repeated experiments (four in total). A constant parameter k in vitro was expected, which enables model validation and primary parameter estimation.
- Day 2: secondary dataset. Findings from the primary dataset were used in optimization of three schemes (CFA‐k, VFA‐k and VFA‐suboptimal). These experiments had an underlying varying k, due to temperature variations between experiments. The data resulting from these high‐SNR experiments could be used to demonstrate the superiority of VFA compared to CFA schemes as well as applicability to experiments with varying model parameters through MC analysis.

In vitro experiments were carried out in an NMR experimental setup in which $B_{1}^{S}$ was verified to be very stable. Thus, the flip angle schemes used in the experiments were optimized with fixed $B_{1}^{S}$, effectively removing the prior from the optimization. A total of five different parameters were estimated, which implies that at least five independently measured scalar data points are needed. Since both EAA and AA are excited and measured simultaneously, three well‐chosen excitations deviating from 0° resulting in six data points are theoretically enough for parameter estimation, although the optimization may in principle show that more are advantageous. The specific flip angle schemes used are presented in Section 4.

#### 3.3.1 dDNP NMR Experimental Setup

A dDNP sample was prepared in which trityl radical Finlandic acid all‐d (1.0 mg, 0.96 μmol) and gadolinium complex gadoteridol (1.3 mg of 50‐μmol/g solution in DMSO) were added to the substrate [1,3‐^13^C_2_] EAA (50.4 mg). The concentration of trityl radical and gadolinium was 20 and 1.4 mM, respectively.

The dDNP [1,3‐^13^C_2_] EAA sample (7.5 mg, 56 μmol) was hyperpolarized in a SpinAligner polarizer (Polarize ApS, Frederiksberg, Denmark) operating at 6.7 T and 1.3 K. After completion of the hyperpolarization buildup, the sample was dissolved in phosphate buffer (5 mL, 50 mM, pH 7.4). From the dissolved sample, 200 μL was injected via an injection line into 100 μL of enzyme solution (CES1, 4 U in phosphate buffer) placed in a 5‐mm Shigemi NMR tube in a Bruker 9.4 T NMR spectrometer operating at 310 K. The enzymatic conversion, the hydrolysis of the ester, was tracked using the optimized flip angle schemes with a TR of 2 s. [1,3‐^13^C_2_] EAA and carboxyl esterase from porcine liver (CES1, EC 3.1.1.1) was purchased from Sigma‐Aldrich.

#### 3.3.2 Data Analysis

The NMR free induction decays were analyzed using MestReNova (Mestrelab Research, Santiago de Compostela, Spain). The corresponding spectra were phase and baseline corrected. The integrals over the EAA and AA spectral peaks are proportional to the transversal magnetization after each excitation and thus to the metabolite concentrations. All data were normalized by the first EAA measure in each dataset. Subsequently, the data of the individual experiments were fitted to each of the two models to estimate the parameters listed in Equations (4) and (7), respectively.

The secondary dataset underwent an additional MC analysis similar to that used in silico as described in Section 3.2. Here, 1000 different realizations of Gaussian noise were added to the experimental data after normalization, resulting in semi‐synthetic data. Subsequently, the data of the individual experiments were fitted to each of the two models, thereby estimating parameters corresponding to those listed in Equations (4) and (7), respectively. The noise was distributed according to $N\left(0,5\cdot 10^{-3}\right)$ and normalized with a factor $\sin\left(\alpha_{0}\right)$ to make the noise comparable across schemes. The level was chosen such that the relative standard deviation of the k parameter estimates was ∼10%. At this noise level, the added noise clearly dominates the existing residuals resulting from measurement noise and model imperfections. An example of a dataset and corresponding fits resulting from normalized empirical data is visualized in Figure 7. In Figure S1 the same dataset is visualized with a realization of the added noise. The same 1000 realizations of noise were used to ease the comparison between experiments.

## 4 Results

### 4.1 In Silico Optimization and Analysis

First, the computationally optimized flip angle schemes are presented, followed by the results of the MC analyses investigating the performance of the schemes in different scenarios.

#### 4.1.1 Optimal Flip Angle Schemes

The CFA‐k scheme was optimized without regularization and resulted in the constant value of 13.5° in six out of the eight different initializations. The remaining were considered outliers based on the optimization not converging, or the FIM being non‐invertible. Infinite variance occurs when the model parameters are underdetermined due to premature saturation of the longitudinal magnetization. As may be expected, the optimized angle is close to the Ernst angle of the metabolic product (15.5° for TR = 2 s), which would give most product signal if the substrate conversion remained constant over multiple TR periods.

**FIGURE 1:**
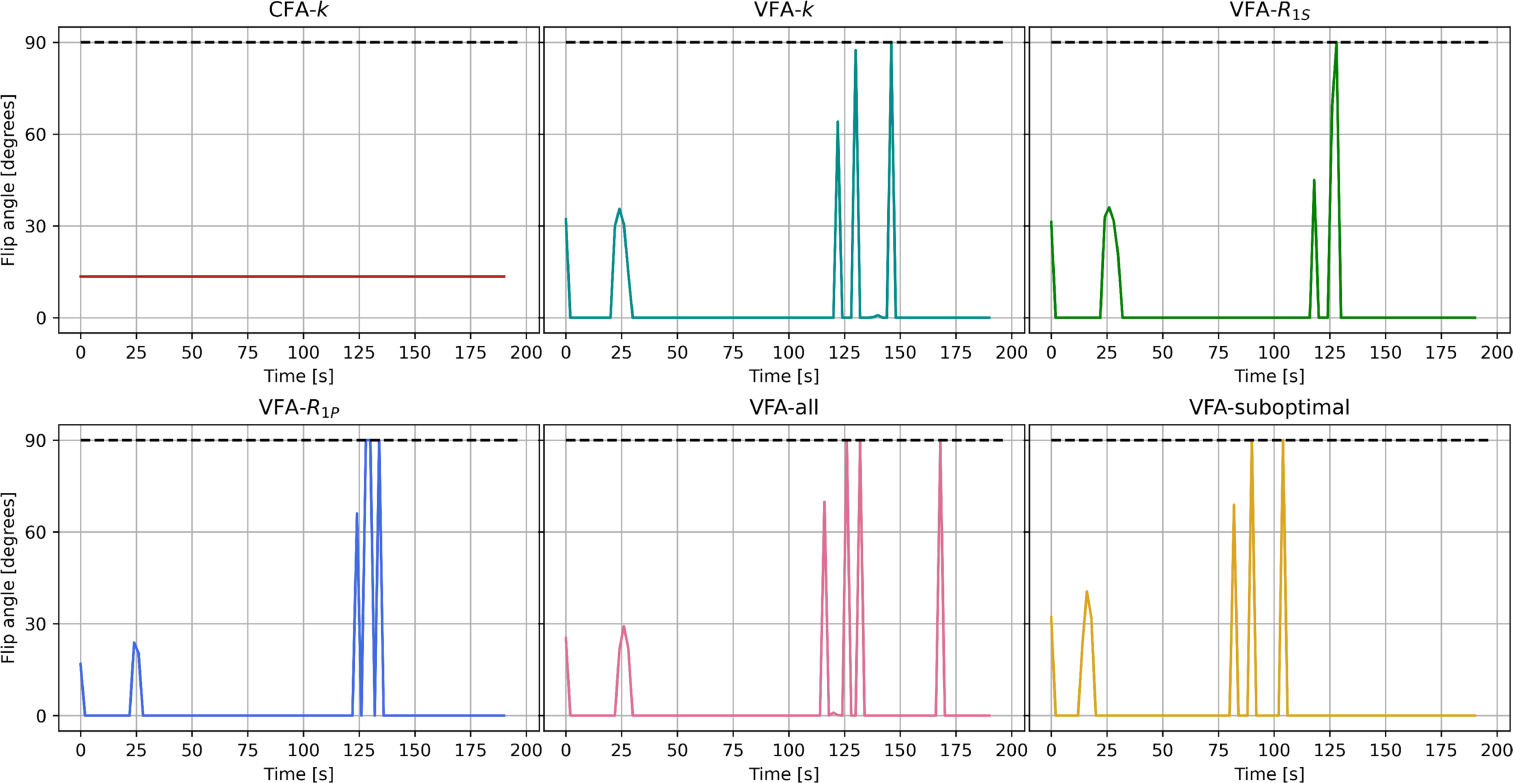
Optimized CFA and VFA schemes where the latter were obtained by minimizing the objective function in Equation (11) supplemented with the regularization from Equation (15). The used nominal parameters and weights are listed in Table 1. The sample interval was TR=2 s. The final series of excitations with large flip angles convert the remaining longitudinal magnetization to signal and can thus be simplified at a cost of limited increased parameter variance, for example, by following the first 90° with one or more consecutive similar pulses that essentially converts remaining magnetization left by RF pulse imperfections to signal.

The VFA schemes were optimized with regularization and initialized 25 times each with a different set of randomly generated flip angles. The regularization was introduced merely to help evaluate convergence of excitation schemes that otherwise fluctuate randomly after magnetization depletion. It proved non‐critical as it only affected the performance insignificantly, which was reflected in minimal effect on the objective function. Example optimizations with different choices of λ are given in Figure S2. Only a few optimizations were outliers. For the remaining schemes, the objective function values were recalculated without regularization. Out of the 25 initializations, 18, 14, 14, 16, and 18 initializations of the five different VFA schemes, respectively, resulted in objective function values within 1% of the scheme with the lowest objective function value. Notably, the first four to eight non‐zero excitations taking place in the beginning of the schemes are almost identical, whereas more variation is seen in later pulses both in terms of timing and flip angle. This confirms that the exact timing and amplitude of later excitations that take place when very little magnetization remains, have low impact on the parameter variances and subsequent objective function value. Common for all of the late pulses is the trend of a gradual increase toward one or more 90° pulses. The same patterns exist for the remaining optimized VFA schemes that resulted in objective function values within 1% of the scheme with the lowest objective function value. Examples are plotted in Figure S3A–E. The schemes that resulted in the lowest objective function values are used in the remaining in silico analysis and are displayed in Figure 1.

VFA‐k, VFA‐$R_{1S}$, VFA‐$R_{1P}$, and VFA‐all have a non‐zero flip angle at t=0 s, two to four flip angles around 25 s later, and some larger flip angles at a much later point in time after 100 s. The VFA‐k and VFA‐$R_{1S}$ schemes are relatively similar, as may be expected, since the model parameters k and $R_{1S}$ affect the longitudinal magnetization of the substrate the exact same way. VFA‐suboptimal is based on nominal parameters that are 1.5 times enlarged. As a result, the late non‐zero flip angles appear at substantially earlier time points.

#### 4.1.2 Scheme‐Dependent Distribution of Parameter Estimates

**FIGURE 2:**
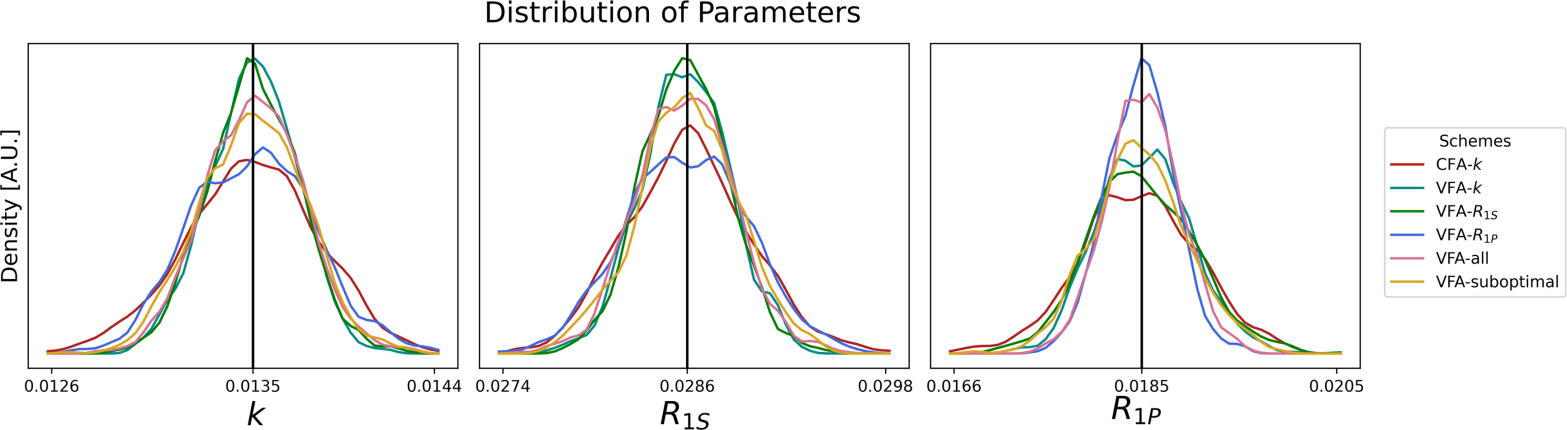
Probability density estimations of the parameter estimates computed by fitting 1000 sets of simulated data. The black vertical lines indicate the ground truth parameter values for k, $R_{1S}$, and $R_{1P}$. Each color corresponds to a different flip angle scheme displayed in Figure 1 and all parameters are fitted using the model without $B_{1}^{S}$.

The kernel density estimates of the parameter distributions resulting from the MC simulations described in Section 3.2 are shown in Figure 2 for the optimized schemes.

First, it is noted that all probability density estimates have a Gaussian distribution and are centered around the nominal parameters marked by black lines. The schemes VFA‐k, VFA‐$R_{1S}$, and VFA‐$R_{1P}$ provided the lower limit of standard deviation achievable for the model parameters k, $R_{1S}$, and $R_{1P}$, respectively. Those lower limits of the rate parameters are 34%, 30% and 43% less than the standard deviations obtained by the optimized CFA‐k scheme. The VFA‐all scheme only resulted in 13%, 12%, and 8% increases in standard deviation when compared to the lower limits, respectively. These relatively small increases indicate that it is possible to reach a reasonable compromise if all rates are of interest.

Furthermore, a separate Bland–Altman analysis was conducted on the k estimates comparing all schemes in a pairwise fashion to assess the accuracy of the results. The analysis results revealed no bias across schemes. A more elaborate description of the analysis can be found in Figure S4 and Section S1.

#### 4.1.3 Robustness to Parameter Variation

**FIGURE 3:**
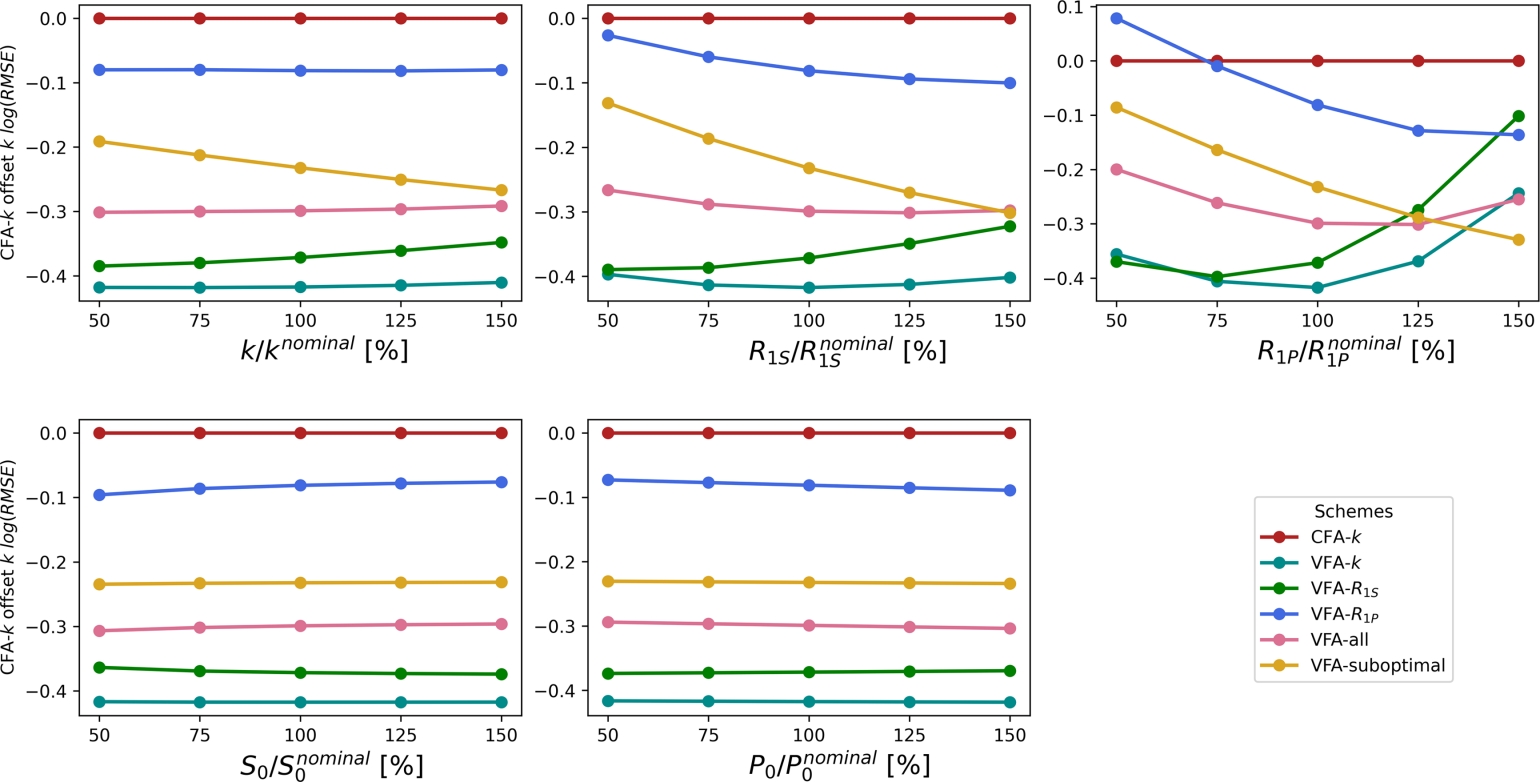
The logarithm of the fitted k RMSE for all schemes with the corresponding values found using the optimized CFA‐k subtracted to use this scheme as a reference. The averages were each based on 1000 realizations of simulated data. The horizontal axis indicates a selection of simulated ground truth parameters deviating from the common nominal parameter values used for flip angle optimization. Data were evaluated for five simulated ground truth parameters ranging from 50% to 150% of their nominal values to verify that measurements conducted with the optimized flip angle schemes are suited for parameter estimation, also when there is substantial mismatch between all actual parameters and the expected parameter values.

The previous results confirmed that better precision of parameter estimates can be achieved with the optimized VFA schemes, when simulations are based on the same nominal parameter values as the scheme was optimized for. In this analysis, the utility of the schemes for measuring parameters deviating from the nominal parameters was also investigated. In Figure 3, the logarithm of the root‐mean‐squared error (RMSE) of the conversion rate estimates k are displayed as a function of model parameter variation in the data simulation. An alternative visualization of these same results is included in Figure S5 with an alternative vertical axis representing relative errors without CFA‐k offset.

It is noted that the VFA schemes almost exclusively determine k better than the CFA‐k scheme across the entire range of model parameters used. Thus, these results indicate that using the VFA schemes more precisely and robustly determine k compared to CFA‐k even when the nominal values are substantially off. The VFA‐k and the similar VFA‐$R_{1S}$ scheme consistently provide the lowest RMSE of the conversion rate k, which would make them the schemes of choice when estimation of the conversion rate is the main priority. The additional RMSE of k found when comparing VFA‐suboptimal to VFA‐k underlines the importance of choosing nominal parameters reasonably for optimization.

#### 4.1.4 $B_{1}^{S}$ Fitting and Robustness to $B_{1}^{S}$ Variation

**FIGURE 4:**
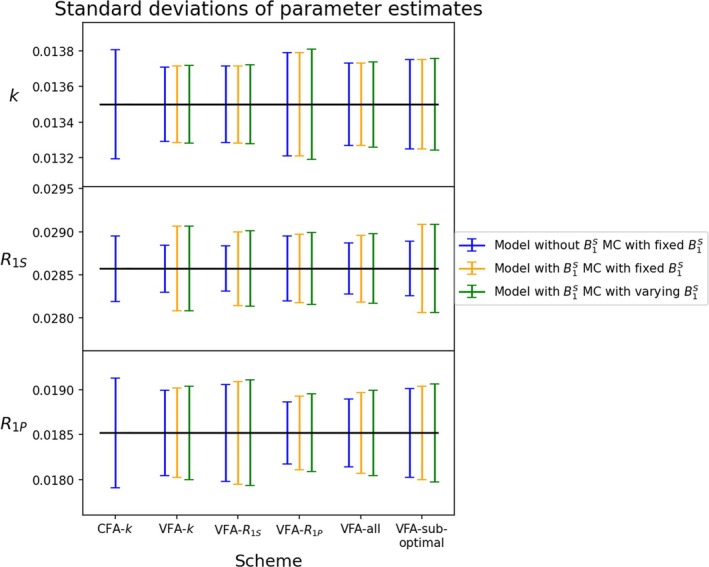
Standard deviation of rate parameters generated through MC simulations utilizing the schemes specified in Table 1. The blue and yellow bars display standard deviations obtained by fixing $B_{1}^{S}=1$ in the MC‐generated data and subsequently excluding (blue) and including (yellow) that same parameter in the model fitting. The green bars display standard deviations obtained by sampling $B_{1}^{S}$ at random from a normal distribution and subsequently including $B_{1}^{S}$ in the fitting. All of the VFA schemes result in lower standard deviations of k estimates compared to CFA schemes.

Moving beyond the assumption of known *B*_1_, it was initially investigated how the uncertainties of parameter estimates were affected by including $B_{1}^{S}$ as a parameter in the model to be fitted. Next, the assumption of non‐varying *B*_1_ was disposed of in the MC simulation by varying the $B_{1}^{S}$ parameter in each realization. The resulting simulations were subsequently fitted with the model including the $B_{1}^{S}$ parameter. All obtained standard deviations are displayed in Figure 4.

The effect of including $B_{1}^{S}$ as a fitting parameter while fixing it in the underlying data realizations can be observed by comparing the blue and yellow error bars. The inclusion of $B_{1}^{S}$ has a minimal effect on the standard deviation of fitted k rates throughout all schemes. However, increased standard deviation is more evident in the remaining rates $R_{1S}$ and $R_{1P}$. Inclusion of an additional parameter increases the degrees of freedom in the model and hence the model variance as expected. The effect of varying $B_{1}^{S}$ and then fitting the data with that parameter can be observed in the green error bars. It was found that the fitted $B_{1}^{S}$ and the ground truth $B_{1}^{S}$ were positively correlated with a Pearson's correlation of 0.996, meaning it is almost perfectly fitted throughout. This explains why the standard deviations of parameters obtained when $B_{1}^{S}$ was included in the fitting were almost identical despite the underlying $B_{1}^{S}$ parameter being varied in one case (green) and not in the other (yellow).

The effect of varying $B_{1}^{S}$ and subsequently fitting the data without that parameter can be observed in the red bars in Figure S6. From these error bars it is clear that the parameter estimates are sensitive to unaccounted flip angle deviations. Interestingly, the variance of the k estimates fitted with CFA‐k and VFA‐all and without fitting $B_{1}^{S}$ (red) closely resembles the ones obtained when $B_{1}^{S}$ was fixed (blue). However, variances of the remaining rates increase substantially and these schemes are therefore only potentially relevant if the conversion rate k is the sole parameter of interest.

### 4.2 In Vitro

#### 4.2.1 Primary In Vitro Experiments

**FIGURE 5:**
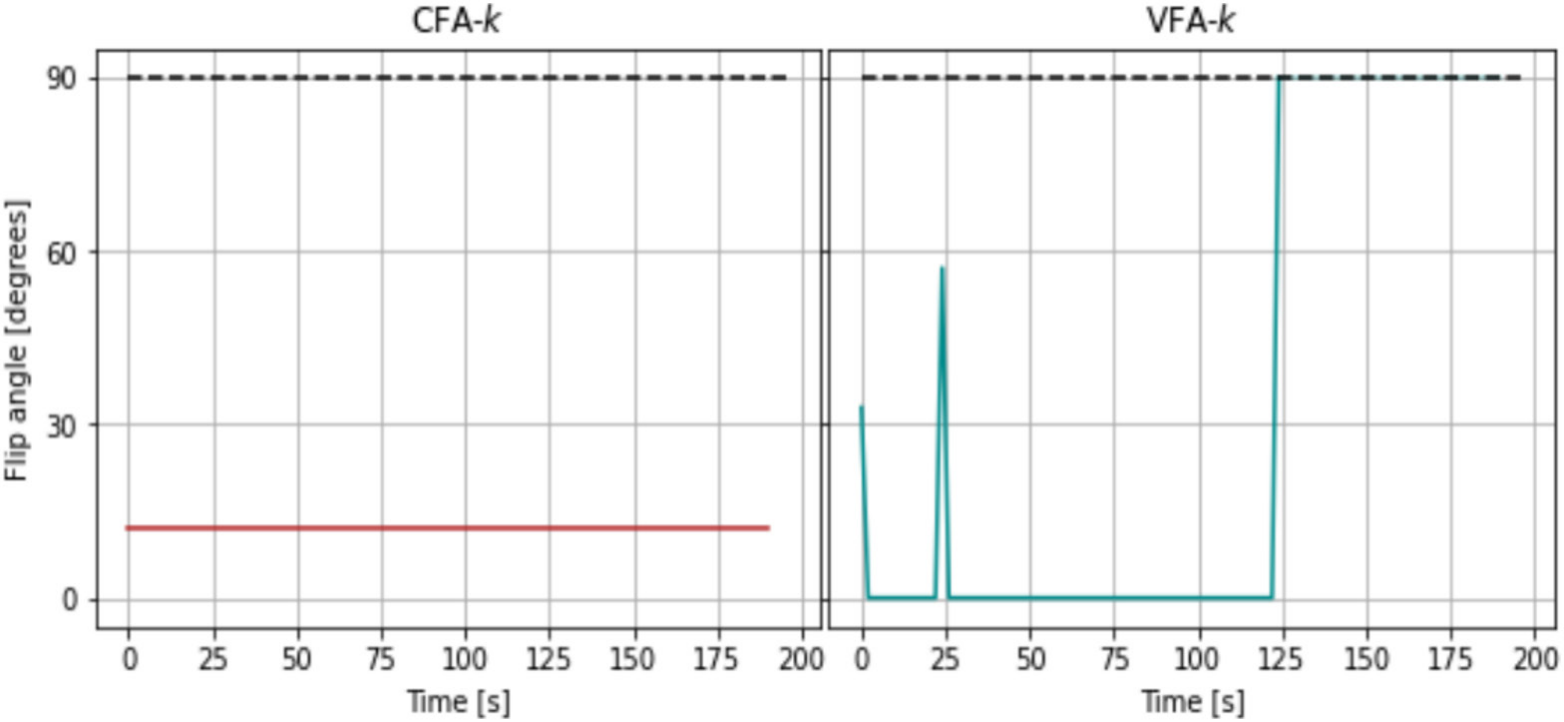
Flip angle schemes used in the primary experiments optimized with weights as listed for CFA‐k and VFA‐k in Table 1 and nominal parameters as mentioned in Section 4.2.1. After the first 90° excitation, no more hyperpolarization is left, if the flip angle is accurate. The remaining flip angles are manually set to 90°, which facilitates evaluation of flip angle accuracy.

In the primary experiments, measurements were performed using a VFA and a CFA scheme, both displayed in Figure 5. The VFA scheme was optimized based on best guess parameters $k=0.01\;s^{-1}$, $R_{1S}=1/\left(36.7\;s\right)$ $R_{1P}=1/\left(51.7\;s\right)$, $S_{0}=1.7$, and $P_{0}=0$. The CFA optimized scheme based on the same parameters would be α=13.4°. However, the CFA experiments were made with a slightly different flip angle of α=12.2°, from an optimization based on a different set of nominal parameters. As these resulting measurements are only used to demonstrate appropriateness of the model, this difference is unimportant.

Note that the 90° pulse that takes place around time t=125 s removes all remaining hyperpolarization and thus none of the subsequent excitations have an impact on the analysis and parameter estimations. This was expected as only three informative measurements of two quantities are needed to estimate up to six parameters. The optimization was initiated with random values and no regularization was used, resulting in randomly fluctuating flip angles after the three needed measurements. However, these were manually set to 90° in the experiment, to measure any magnetization that may remain due to flip angle inaccuracies.

Initial fitting of the primary data sampled using the VFA‐k scheme and the model including $B_{1}^{S}$ consistently resulted in $B_{1}^{S}\approx 0.85$. In the subsequent analysis, all flip angles were corrected by multiplication with this factor to compensate for the experimentally determined inaccuracy. The resulting parameters are displayed in Table 2.

**TABLE 2:** Parameters fitted to the data obtained from the primary experiments utilizing both models with and without $B_{1}^{S}$.

Scheme	$B_{1}^{S}$ fit	Dataset	*k*	$R_{1S}$	$R_{1P}$	$B_{1}^{S}$
CFA‐*k*		D1	0.0130	0.0285	0.0160	
CFA‐*k*		D2	0.0136	0.0293	0.0192	
VFA‐*k*		D3	0.0137	0.0293	0.0227	
VFA‐*k*	x	D3	0.0137	0.0295	0.0229	0.993
VFA‐*k*		D4	0.0137	0.0274	0.0173	
VFA‐*k*	x	D4	0.0137	0.0273	0.0172	1.007

All experiments were performed using a subsample of the same enzyme solution to obtain identical conversion rates. Both models reproduced the observed data reliably in all four experiments and with small maximum residual compared to the modeled signal maximum (< 1%). Furthermore, the estimates of the rates were very consistent across experiments and across the flip angle schemes. This provides evidence that the models accurately describe the dynamics of the magnetization in the experiment. Hence, it was demonstrated that using these models for in silico investigations is warranted and that the rates derived from the high SNR data can be trusted and treated as ground truth, within the limits of the model.

#### 4.2.2 Secondary In Vitro Experiments

**FIGURE 6:**
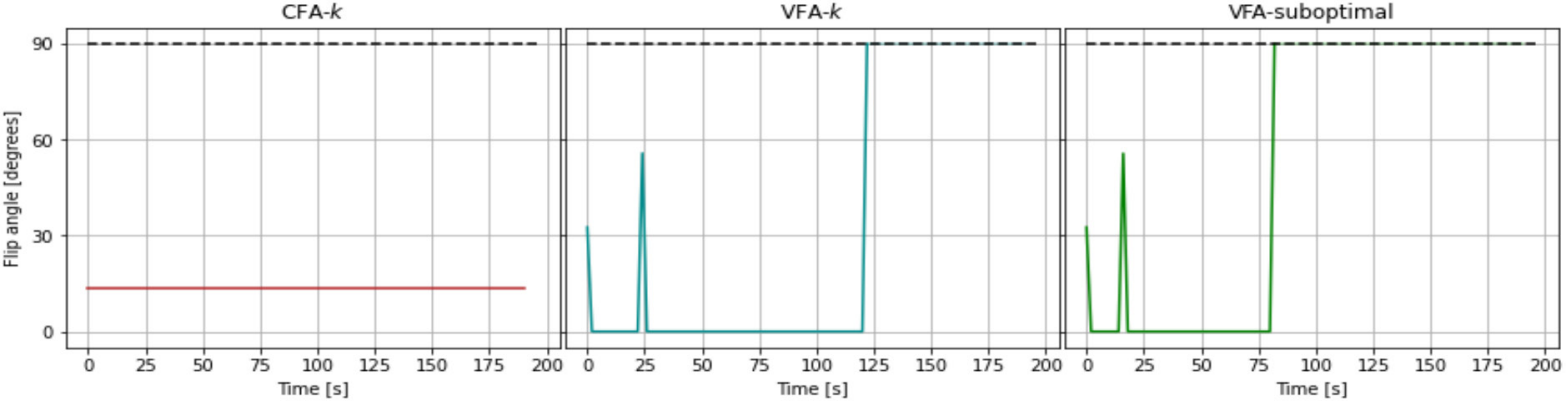
Flip angle schemes used in the secondary experiments optimized with specifics as listed for CFA‐k, VFA‐k, and VFA‐suboptimal in Table 1. After the first 90° excitation, no more hyperpolarization is left if the flip angles are accurate. The remaining flip angles are manually set to 90°, in case they are not.

Based on the new knowledge about parameter values from the primary experiments, three new optimized schemes were determined without the $B_{1}^{S}$ prior. These were optimized with the specifics listed for CFA‐k, VFA‐k, and VFA‐suboptimal in Table 1. The CFA optimization yielded flip angles of 13.4° and all schemes are displayed in Figure 6. Similar to the schemes optimized for the primary experiment, all flip angles after the first 90° pulse were manually set to 90°. Furthermore, since the effective $B_{1}^{S}$ in the experimental setup was now known, all flip angles were corrected by division with 0.85 in the following experiments. The robustness of the estimated conversion rate k to the ground truth parameter variation was investigated using MC simulation and gave similar results as the initial in silico robustness analysis displayed in Figure 3.

**FIGURE 7:**
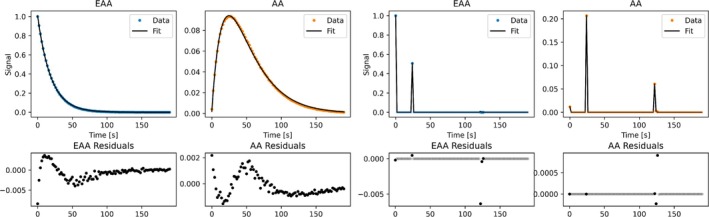
Upper row: Dots represent experimentally obtained signal values proportional to transversal magnetization, normalized by the first point of the EAA trajectory. The black lines are the corresponding fits of the substrate EAA and product AA. The transparent dots represent points that were measured, but not included in the data fitting, as no excitation occurred. Lower row: Residuals resulting from the fits, which notably stay below 1% of the maximum substrate signal. Results in the two leftmost columns were obtained using a CFA‐*k* scheme, while the two rightmost columns used a VFA‐*k* scheme.

Six experiments were carried out using each of the schemes displayed in Figure 6 twice. Examples of the data, their fits and residuals are displayed for two experiments using CFA‐k and VFA‐k respectively in Figure 7.

Similar to the primary experiment, the models fitted the data well with small residuals, indicating that the model describes the underlying process well and is warranted. Thermal noise is expected in these types of measurements, resulting in residuals with a normal distribution. However, the residuals clearly deviate from what would be expected from a pure thermal noise distribution, which means that some signal features are not captured by the model. Such systematic residuals are common [26, 27, 28], and the experiments exemplify the significance of such model imperfections.

**FIGURE 8:**
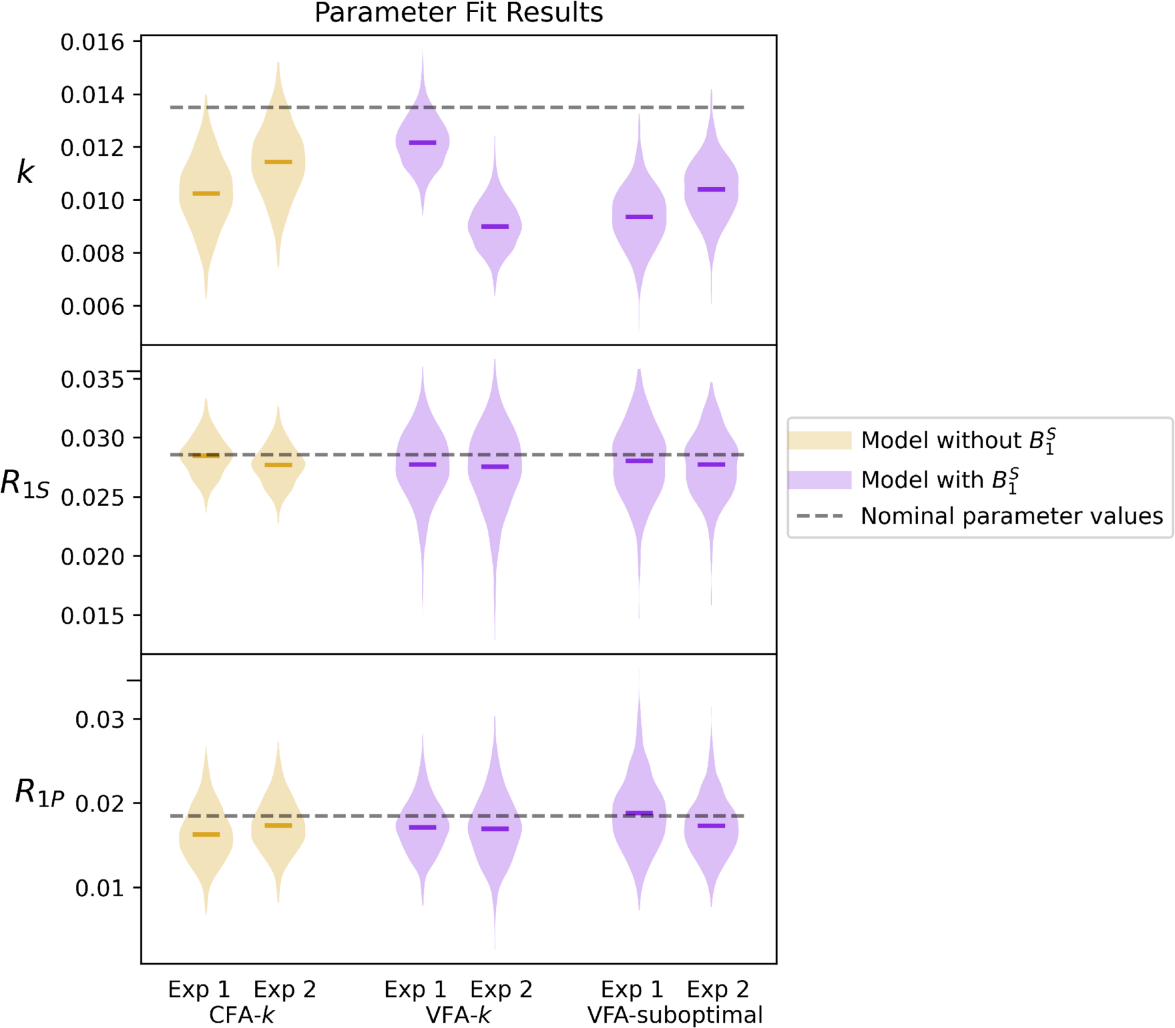
The colored lines mark the value of the fitted parameters in the individual datasets (repeats Exp1 and Exp2). The horizontal axis indicates groups of repeated experiments with common schemes for data collection. The shaded areas represent the probability density estimation of the parameter estimates obtained through MC simulation, and the colors indicate the model used to fit the data. The parameters obtained through use of the CFA‐k scheme were fitted with the model excluding the $B_{1}^{S}$ parameter, while the parameters obtained through use of the VFA schemes were fitted with the model including the $B_{1}^{S}$ parameter. The gray dashed lines indicate the nominal parameters used in the sequence optimization.

Since there was temperature variation between experiments (not measured), the conversion rates were expected to differ. The datasets sampled with the CFA‐k scheme were fitted with the model excluding $B_{1}^{S}$ since use of CFAs makes $B_{1}^{S}$ and $T_{1}$ effects indistinguishable. The remaining datasets were fitted with the model including $B_{1}^{S}$. The parameters estimated from model fitting to each individual dataset are marked by horizontal lines in Figure 8. A MC analysis was conducted in which parameters were fitted to semi‐synthetic data generated by the addition of Gaussian noise to experimental data. Thereby repeated experiments were simulated only differing in the realization of simulated thermal noise present. The shaded areas in Figure 8 show the distribution of the parameter estimates obtained when fitting the model without $B_{1}^{S}$ to the CFA‐k datasets and the model with $B_{1}^{S}$ to the remaining VFA scheme datasets. The standard deviations of the k estimates obtained using the VFA schemes were substantially lower compared to that of the CFA‐k scheme. Note that this decrease occurred despite the VFA analysis involving fitting of an additional model parameter $B_{1}^{S}$. The average standard deviations of estimates of k obtained with VFA‐k and VFA‐suboptimal respectively were 32% and 15% less when compared to CFA‐k.

The estimated conversion rate k varied as expected due to the temperature variation. The relaxation rates $R_{1S}$ and $R_{1P}$ remained very consistent across experiments and had estimated values similar to those obtained in the primary experiments. The estimated $B_{1}^{S}$ values were close to 1.0 as expected, since the flip angles had been corrected prior to these experiments.

All results indicated a smaller standard deviation of the conversion rates k when estimated using VFA schemes compared to the CFA scheme, with the smallest variation obtained using VFA‐k as expected. These results indicate that despite an imperfect model fit and deviation from nominal parameters, the determined VFA schemes outperform the CFA schemes.

## 5 Discussion

Based on existing approaches, we advanced a method for determining VFA schemes that provide estimates of the pharmacokinetic parameters with minimal variance. It was shown computationally that the determined VFA schemes outperformed even optimized CFA schemes for parameter estimation. Additionally, the simulations demonstrated robustness to underlying parameter variation including $B_{1}^{S}$ when incorporated in the experimental design and model fitting. However, for the determined schemes to be effective in an experimental setting, prior assumptions about the system and realistic parameter ranges have to be made. Such information is typically available from theory, knowledge about related systems, or pilot experiments aimed at model building or model validation. The optimization of flip angle schemes involves trade‐offs, for example, reflected in chosen parameter weights that prioritize parameter variances, or choice of priors that affects the computational load. Lastly, in vitro investigations exemplified the methodology in a system where the model was demonstrated to be suitable. In contrast, severe model imperfections can compromise the value of parameter estimates, cause residuals to be non‐Gaussian and violate assumptions when optimizing the flip angle schemes and fitting the data to kinetic models.

### 5.1 Effects of Parameter Weighting Choices

Before optimization, a set of weights need to be chosen to assign relative importance of the parameter variances in the objective function. The VFA‐all scheme was one example of a scheme that utilized a combination of weights and was compared to the lower limits of achievable standard deviations of model rates. It was designed to express approximately similar interest in all parameters. However, since it is not clear a priori how exactly the individual weights affect the uncertainties of the resulting model parameters, the weights may advantageously be iteratively adjusted to obtain the preferred balance between parameter uncertainties in MC simulations. Increasing the weight of one parameter may cause a reduction in its standard deviation but may also increase the standard deviation of other parameters. Importantly, the VFA‐all scheme offers a good starting point for iterative weight adjustment, and the VFA‐c schemes reveal the limits of obtainable reliability of each parameter c.

### 5.2 Additional Robustness to Parameter Variation

Expectations of actual parameter values (priors) are always used when designing measurements, for example, implicitly via the choice of measurement equipment. In the present case, specific priors enter explicitly. Although robustness to the prior was demonstrated for a wide range of variation, it is clear that there is a limit. Ideally, an implementation taking general priors on all the model parameters to be estimated as inputs, would provide schemes designed to perform well over the specified parameter ranges. This approach was demonstrated for the parameter $B_{1}^{S}$ known to vary considerably in MRI and thus potentially making the choice of a single nominal value problematic. It is beyond the scope of this work, but major uncertainty of several parameters can readily be accommodated by altering the objective function to integrate over a multi‐variate distribution prior $p_{0}\left(\theta \right)$:

(16) $$\min_{\alpha }\;\int_{\;\theta }f_{L}\left(ℐ\left(\theta ,\alpha \right)\right)\cdot p_{0}\left(\theta \right)d\theta$$

The challenge of selecting nominal values is limited in the frequent case where deviations from known parameter values are of interest. Determining how a disease, treatment, or intervention affects known parameter values is a typical experimental objective, for example. In this case, optimizing the measurement sensitivity to small changes from known parameter values is often crucial. The outlined method does exactly that in its simplest form but can furthermore incorporate pronounced uncertainty via priors.

### 5.3 $B_{1}^{S}$ Fitting

Flip angle uncertainty is sometimes used as an argument against VFA schemes, as flip angle miscalibration can cause severe SNR loss. We demonstrated that robust schemes can be designed and that these allow for precise and accurate $B_{1}^{S}$ estimation. The results of the MC analysis shown in Figure S6 showed that the data obtained with CFA‐k and VFA‐all schemes, fitted with the model without $B_{1}^{S}$, resulted in reasonable k estimates despite underlying $B_{1}^{S}$ variation (red), thus outperforming the remaining VFA schemes. Scatter plots of MC parameter estimates in Figure S7 reveal that CFA‐k and VFA‐all provide k‐estimates that are relatively insensitive to $B_{1}^{S}$ unlike the remaining optimized schemes, where estimates of k systematically depend on the size of $B_{1}^{S}$. For this reason, fitting $B_{1}^{S}$ independently was necessary for these schemes to result in reliable model parameter estimates. However, if an optimized CFA scheme is used for data collection and $B_{1}^{S}$ therefore cannot be fitted, k can be estimated with a reasonably low uncertainty, which requires that no model parameters are fixed during fitting. As a result, the relaxation parameter estimates will be very inaccurate and correlated with the $B_{1}^{S}$ estimates. If alternatively a VFA scheme is used and $B_{1}^{S}$ is fitted, much smaller parameter uncertainties can be achieved, while all parameters are estimated without bias.

### 5.4 System Features Not Captured by the Model

The data fitting results demonstrate a good fit of the model to the data as seen in Figure 7. However, the fit has residuals that clearly deviate from the normal distribution expected from thermal noise alone and show a small but clear systematic error. Such model imperfections are typical and may be due to multiple underlying reasons. First, the model used in this work relies on two known simplifying assumptions. The enzymatic reaction is assumed unidirectional although the product can be converted back into substrate. This back‐conversion is known to be very limited and is thus disregarded. Furthermore, the conversion rate k is assumed constant although it is known to vary with the total concentration of the substrate, being the sum of unpolarized and hyperpolarized substrate. During the course of the in vitro experiment, this total concentration of substrate decreases due to its conversion to product, and as a result, the conversion rate k gradually decreases. This is a limited concern in vivo as some substrate and product is already present, and the perturbation of the chemical equilibrium is limited. Introducing more parameters can improve the residuals of the model fit, as the model is known to be simplified. However, including more parameters into a model may increase the variances of the parameter estimates and limit their practical value, even if the parameters are slightly better defined in a more accurate model. Second, imperfections and inconsistencies in the *B*_1_ field strength are known to be present in the spectrometer. The sample is typically not fully emerged in the coil due to physical constraints. Convection also takes place, resulting in the different parts of the solution experiencing a range of *B*_1_ field strengths over time. These effects were ameliorated in practice by multiplication of a factor $B_{1}^{S}$ representing the difference between the calibrated flip angle and an effective flip angle. The resulting model deviations were minor as reflected in the residuals.

### 5.5 Implications of Non‐Gaussian Residuals

The equations used for optimization are based on an assumption of a Gaussian distribution of noise although the fitting resulted in a different distribution of the residuals. Thus, the schemes are optimized based on a violated assumption. The model parameter estimates resulting from the in vitro experiments showed that despite the schemes only being optimized for a nominal set of expected parameter values, the k estimates were robust and repeatability was achieved across multiple individual experiments.

In the MC simulations based on semi‐synthetic data, the non‐random residuals reflecting model imperfections were equal across simulated repeated measurements. This constitutes a worst‐case scenario, as stochastic effects will realistically make residuals from model imperfections more random across repeated measurements, which the optimized schemes account for by design. Despite representing the worst case, no VFA performance loss was observed for any of the non‐random residuals observed experimentally. This is reassuring but not surprising, considering that the added random noise dominated the non‐random residuals of the high‐SNR experimental data. The resulting simulated signal characteristics are realistic, since the proposed sequence optimization is relevant only when SNR is a limiting factor. The very high SNR of the demonstration experiments was needed to make the thermal noise insignificant, but this condition is unrealistic in relevant use cases in vivo, for example.

### 5.6 Generalization to Other Systems, Metabolite‐Specific Excitation and Imaging

While this work focused on a unidirectional metabolic conversion model, the described methodology can be directly generalized to other conversion schemes, where the requirements are met. The approach can relatively simply be extended to include additional metabolites and rates in the model to match a different system of interest. In addition, metabolite‐specific excitation can be incorporated in the theoretical framework by introducing a fixed or optimizable flip angle scaling factor for each metabolite. Alternatively, each flip angle in the α vector can be replaced with an array of independent flip angles (one per metabolite excited independently), thus replacing α with a matrix of at least twice as many parameters to optimize. The experimental scheme must then also be updated and use one or more correspondingly shaped RF pulses, which may be challenging depending on the flexibility of the sequence, the proximity of metabolite resonance frequencies, and on the inhomogeneities of $B_{0}$ and $B_{1}$.

This work included the fitting of $B_{1}^{S}$, and robustness to its variation was ensured through a wide prior distribution on $B_{1}^{S}$. This is of particular importance since the *B*_1_ field can be inhomogeneous and unknown, and its effect on the excitation pulses may affect parameter estimates. However, to apply the presented optimization strategies to in vivo experiments, additional steps may have to be taken such as updating the FIM calculation to assume Rician measurement noise distribution typically assumed for MRI when images are not made real‐valued by subtraction of an estimated phase. However, the high SNR associated with few large‐angle excitations rather than many small‐angle excitations can make the assumption of Gaussian noise valid, which also simplifies analysis immensely as it is non‐trivial to analyze low‐SNR data with Rician noise since RMSE parameter estimates may become biased. Including bolus characteristics through an input function may also be needed [29]. This requires additional model parameters to be fitted, and the resulting model may contain correlated parameters. An optimized acquisition strategy may again help to disentangle them. Furthermore, additional factors such as slice profile effects in MRI that cause intra‐voxel variation of the excitation angle may require model adaptations needed for both the parameter estimation and the excitation scheme optimization.

### 5.7 Implications of Design Choices

The flip angle schemes determined and verified in this work deviate substantially from what is commonly used and what has previously been found in related studies, particularly with regard to the small number of non‐zero excitation angles. Our results are theoretically expected since the limited magnetization is most efficiently spent at relatively few time points where the measurements are most informative. Similar conclusions are reached in other contexts, for example, by Jones et al. [30].

Importantly, the regularization is not responsible for the sparsity of initial excitations as demonstrated by Figure S2. Also, sparsity is not enforced in other ways. The limited pool of hyperpolarized magnetization is responsible, as the measured information about a specific set of parameters cannot be improved by diluting the signal over many measurements. Rather the opposite is true, as measurements at specific time points are more informative than others. The optimal timing depends on $B_{1}^{S}$ and the time constants of the studied system. When $B_{1}^{S}$ is not assumed known, excitations are therefore preferably more distributed in time, as also observed in the optimized schemes.

The general approach used in this work was based on the studies by Maidens et al. [10, 11]. Numerous differences in design choices contribute to the discrepancies in this study and in their work. First, the choice of model is fundamental and dependent on the system of interest. The work of Maidens et al. focused on in vivo systems and included an arterial input function requiring the addition of several extra parameters making that model a two‐site model as opposed to our one‐site model. Given that MRI was the target of Maidens et al., the noise was assumed Rician instead of Gaussian. Lastly, different choices were made with regards to the parameters optimized for, the optimization criteria, regularization and nominal parameters. Our work, on the other hand, included the $B_{1}^{S}$ parameter to account for flip angle deviations that may remove the advantages of VFA schemes, when not accounted for.

### 5.8 Recommended Practical Procedure

**FIGURE 9:**
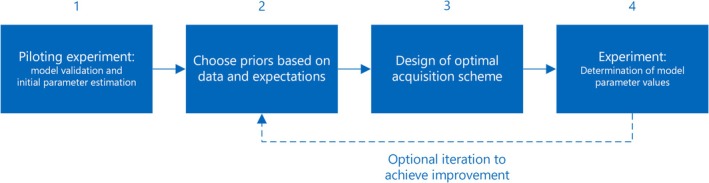
Flow chart outlining a practical workflow involving VFA optimization to obtain maximally reliable parameter estimates. (1) Pilot experiments aim to build/validate the model and provide rough parameter estimates. (2) These and other available information inform the choice of nominal parameters or priors as input for flip angle optimization (3). The optimized scheme is subsequently used in experiments (4), and the resulting data are analyzed to provide new and more reliable parameter estimates. These can optionally feed back into (2), thus repeating part of the procedure from a more informed standpoint. The parameter weights in the objective function may also be adjusted, for example, iteratively based on MC simulation, to consistently perform the most informative measurements. This option is not shown in the chart but described in Section 5.1.

A workflow implementing the proposed methods is illustrated in Figure 9. A prerequisite for the optimization procedure is that the model for the data is appropriate such that the parameter estimates describe the underlying dynamic process. Pilot experiments may thus be needed and for the current system, these could for example be conducted using CFA schemes with small flip angles around the expected Ernst angles of the metabolic products, since excitation at the product Ernst angle balances metabolic conversion against polarization losses from relaxation and measurement. Such experiments and the subsequent data fitting will also provide rough parameter estimates given the $B_{1}^{S}$ is assumed known. While optimized VFA schemes improve the SNR of the individual measurements, and the sensitivity to the chosen parameters, they are not necessarily good for model validation as the number of measurements may not exceed the number of model parameters. Importantly, the gains of optimized VFA schemes over short‐TR CFA do not come from longer time between excitations alone. Increasing TR for CFA schemes will increase conversion between excitations and may increase the product signal and the gained information up to the point where the TR is too long to temporally resolve the system dynamics. However, the full benefit of the proposed schemes comes from spending the optimum amount of magnetization at times where the measurements are maximally informative, in accordance with the presented mathematical description. A calculation supplementing the described in silico analysis and being based on the same parameter choices, showed that the best CFA scheme for measuring the metabolic rate k optimized with respect to repetition time and flip angle resulted in 17% more uncertainty of the k estimate than the optimized VFA scheme. Once a model is validated, CFA is outperformed by optimized VFA schemes based on nominal values or priors incorporating all available information.

### 5.9 Future Work

This work paves the way for adaptive MR measurements with (real‐time) adjustment of the data acquisition using the information available, that is, using both prior information and already acquired data. For the examined model and fixed $B_{1}^{S}$, the optimized schemes consist of three measurements including one at bolus arrival. The measurement at this time provides information about the two parameters $S_{0}$ and $P_{0}$, and the ratio between the two could have implications for how the magnetization is used most efficiently for the remaining excitations. However, the current implementation is too slow to optimize an updated flip angle scheme real‐time. Currently, the optimization process with multi‐start takes several hours using a 3‐GHz 32 core processor, for example. Machine learning could be a useful tool to speed up the process by replacing time critical function evaluations with surrogate estimates fitted via machine learning approaches.

## 6 Conclusion

In this work, we formulated a dynamic model describing unidirectional enzyme‐driven conversion in a hyperpolarized NMR experimental setting, and optimization was used to determine constant and VFA schemes that minimize a weighted uncertainty of model parameters to be estimated.

Through MC simulations, it was computationally validated that compared to CFA schemes, the optimized VFA schemes provide more reliable estimates of model parameters. For the metabolic rate parameter, k, specifically, a 34% reduction in standard deviation was achieved. Furthermore, this reduction was stable even when the model parameters used for simulation deviated from the nominal values. This implies that optimized VFA schemes outperform CFA schemes, even when model parameters are not known accurately in advance. Due to correlation of the model parameter estimates, it was concluded that fitting the $B_{1}^{S}$ parameter is essential for estimating the remaining model parameters, when the *B*_1_ field strength is spatially varying or not accurately known.

In vitro experiments demonstrated that the model used in silico was suited as model parameters could reliably be reproduced using different flip angle schemes. Through the analysis of semi‐synthetic data, it was demonstrated that an improvement of 32% in the standard deviation of k could be obtained using VFA schemes compared to an optimized CFA scheme.

Given that the *B*_1_ field strength is included in the model or is known accurately in advance, we conclude that VFA schemes consistently outperform CFA schemes by providing more precise metabolic rate estimates in MR experiments.

## Author Contributions

Conceptualization, developing, and designing the study: all authors. Acquisition of experimental data: P.R.J. Analysis and interpretation of data: M.F.G., L.G.H., K.H.M., and R.B.O. Script programming: M.F.G. and K.H.M. Resources: J.H.A.‐L. Drafting manuscript and visualization of results: M.F.G. Supervision: L.G.H., R.B.O., K.H.M., P.R.J., and J.H.A.‐L. Funding acquisition: J.H.A.‐L. Reviewing and editing of manuscript: all authors.

## Conflicts of Interest

Jan Henrik Ardenkjaer‐Larsen is the owner of Polarize Aps that manufactures equipment for hyperpolarization.

## Supporting Information

Additional supporting information can be found in the online version of the article at the publisher's website.

## Supporting information


**Figure S1:** Realization of semi‐synthetic data.
**Figure S2:** Effects of changing size of λ.
**Figure S3:** Optimal VFA schemes from multistart.
**Figure S4:** Bland–Altman analysis.
**Section S1:** Description of Bland–Altman analysis results.
**Figure S5:** Relative error.
**Figure S6:** $B_{1}^{S}$ fitting with varying underlying $B_{1}^{S}$.
**Figure S7:** Scatter plot of parameter estimates.

## Data Availability

The functions and code used for the in silico investigation are available at https://github.com/MarieGarnaes/Optimal‐VFA‐Schemes.
