# Supplementary material for: Optimal Variable Flip Angle Schemes for Hyperpolarized MR Kinetic Modeling Robust to RF Field Variations
Source: NMR Biomed. 2025 Oct 11;38(11):e70151. doi: 10.1002/nbm.70151 (PMC12514949; doi:10.1002/nbm.70151)
Supplement: Supplementary file 1 — Figure S1: Realization of semi‐synthetic data. Figure S2: Effects of changing size of λ. Figure S3: Optimal VFA schemes from multistart. Figure S4: Bland–Altman analysis. Section S1: Description of Bland–Altman analysis results. Figure S5: Relative error. Figure S6: B1S fitting with varying underlying B1S. Figure S7: Scatter plot of parameter estimates. [file NBM-38-e70151-s001.docx]

**Supporting Information**

**Figure S1:** Realization of semi-synthetic data

**Figure S2:** Effects of changing size of λ

**Figure S3:** Optimal VFA schemes from multistart

**Figure S4:** Bland-Altman analysis

**Section S1:** Description of Bland-Altman analysis results

**Figure S5:** Relative error

**Figure S6:** $B_{1}^{S}$ fitting with varying underlying $B_{1}^{S}$

**Figure S7:** Scatter plot of parameter estimates


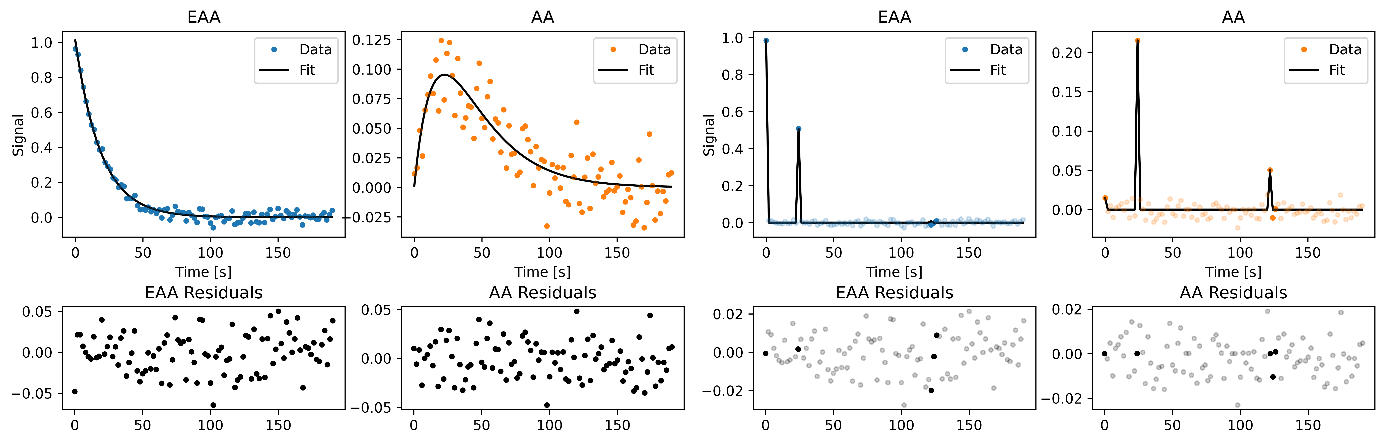


**Figure S1:** Upper row: Dots represent semi-synthetic data, which is the same data as presented in Figure 7, but with Gaussian noise added. The black lines are the corresponding fits of the substrate EAA and product AA. The transparent dots represent measured noise that is not included in the data fitting, since no excitation occurred. Lower row: Residuals resulting from the fits, which notably stay below 6% of the maximum substrate signal. Results in the two leftmost columns were obtained using a CFA-k scheme, while the two rightmost columns used a VFA-k scheme.

| A) | 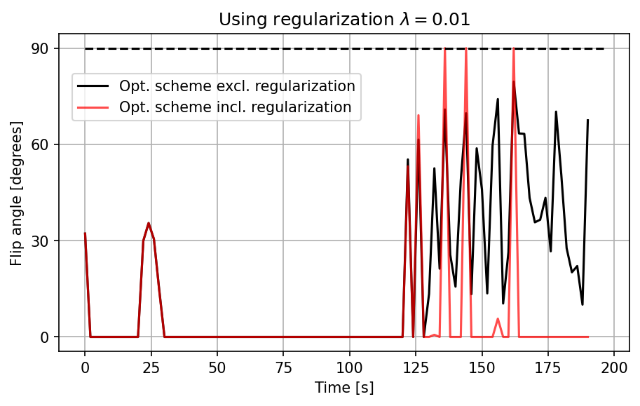 | B) | 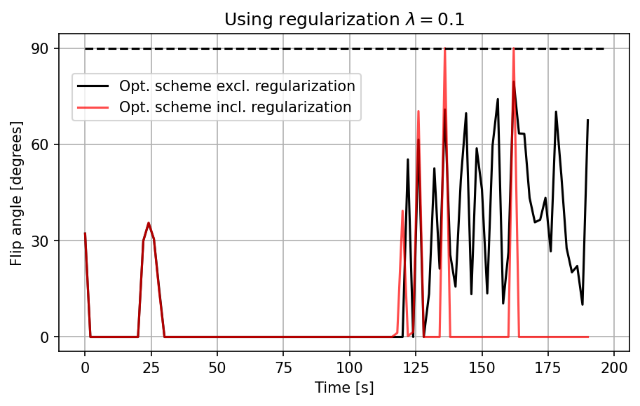 |
| --- | --- | --- | --- |
| C) | 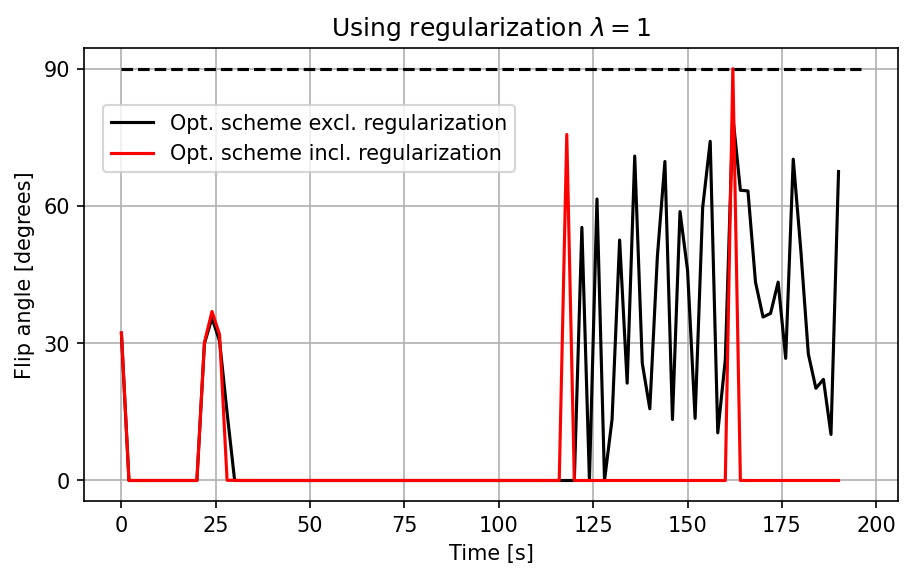 |  |  |

**Figure S2:** VFA-*k* schemes optimized applying different amounts of regularization in red by changing sizes of λ as indicated in the title. The black graphs depict optimized VFA-*k* schemes excluding regularization for reference.

| A) | 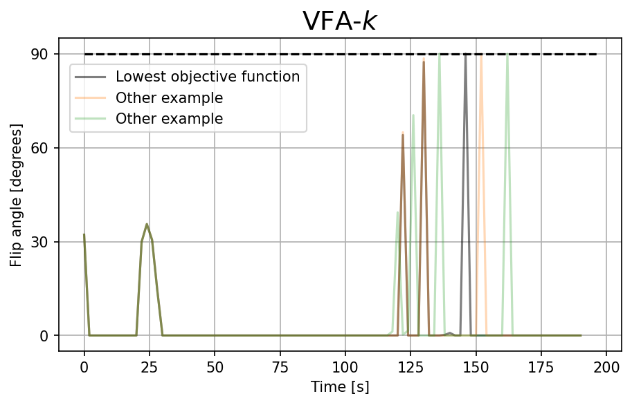 | B) | 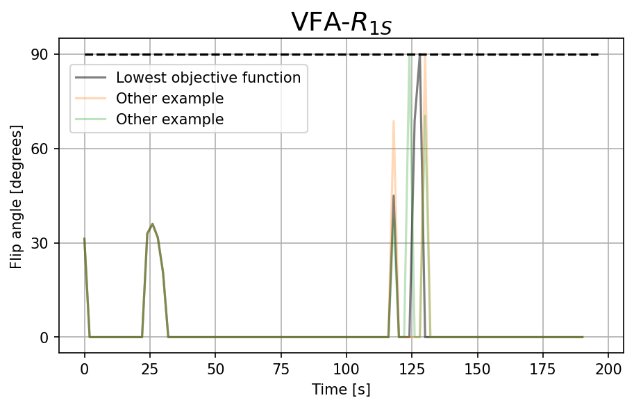 |
| --- | --- | --- | --- |
| C) | 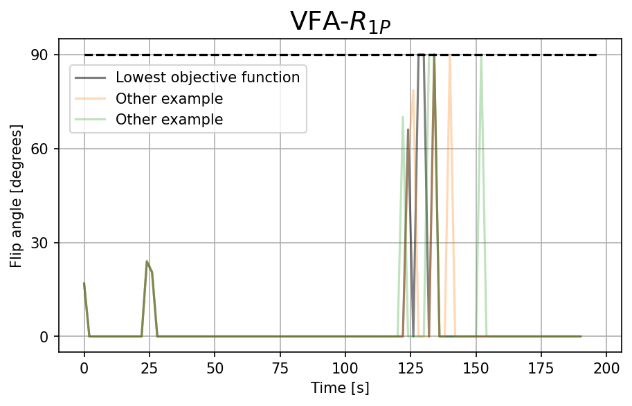 | D) | 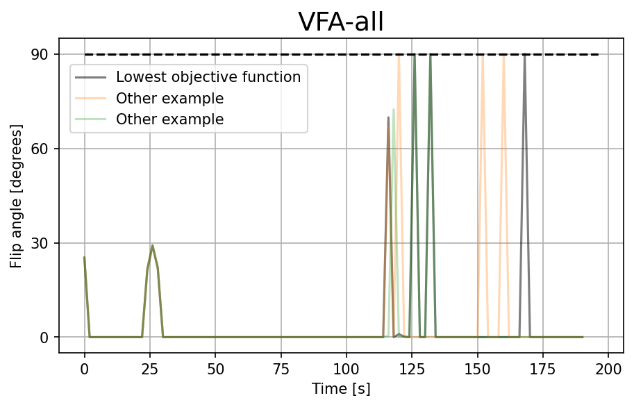 |
| E) | 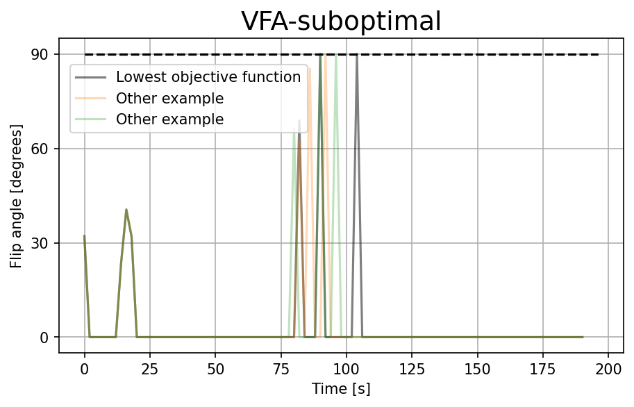 |  |  |

**Figure S3:** Figures A-E display optimized VFA schemes resulting from a number of random initializations. The scheme displayed in black results in the lowest objective function value when regularization is excluded. The remaining schemes marked with transparent colors result in objective function values less than $1\%$ larger than the lowest obtained. In practice, the final series of $90^{\circ}$ pulses can be truncated or the pulse timings changed with insignificant increases in the parameter variances.


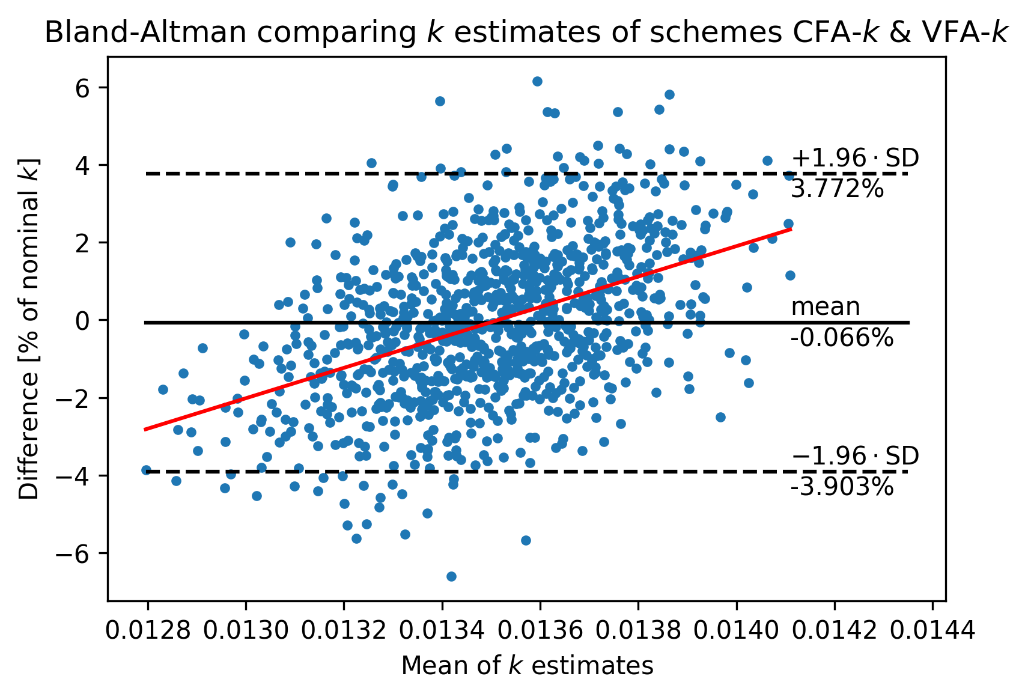


**Figure S4:** Bland-Altman plot comparing estimates of *k* using the schemes CFA-*k* and VFA-*k*, respectively. The blue dots represent pairs of *k* estimates from MC simulations, only differing by the excitation scheme used. The horizontal axis indicates the mean of the estimates and the vertical axis indicates the differences between the estimates in percentage of the nominal *k* value. The full black line marks the mean difference between the schemes and the dashed lines indicate limits of agreement. The red line is a fitted linear slope.

**Section S1:**

A total of 15 Bland-Altman plots were generated to pairwise compare *k*-estimates of all schemes. The results of all comparisons are collectively described in this section, but only one plot is visualized in Figure S3 above as an example. All plots displayed mean difference values of <0.15%, with narrow limits of agreement of <5%. Therefore, it was concluded that no significant bias was found between *k* estimates originating from the various schemes. Furthermore, the variability of dots representing pairs of *k* estimates was relatively consistent but increasing or decreasing trends were observed for most plots. These linearly fitted slopes were all significant according to t-tests except the VFA-*k* & VFA-$R_{1S}$ comparison. The very different variances of the *k* estimates can be the cause of these linear trends^[[1]](#footnote-1)^. In fact, this explanation is consistent with all the Bland-Altman plots: High-variance parameter estimates minus low-variance parameters estimates (e.g. CFA-*k* vs. VFA-*k* as displayed in the figure) results in a positive slope while low-variance parameter estimates minus high-variance parameters estimates (e.g. VFA-*k* vs. VFA-$R_{1P}$) results in a negative slope.


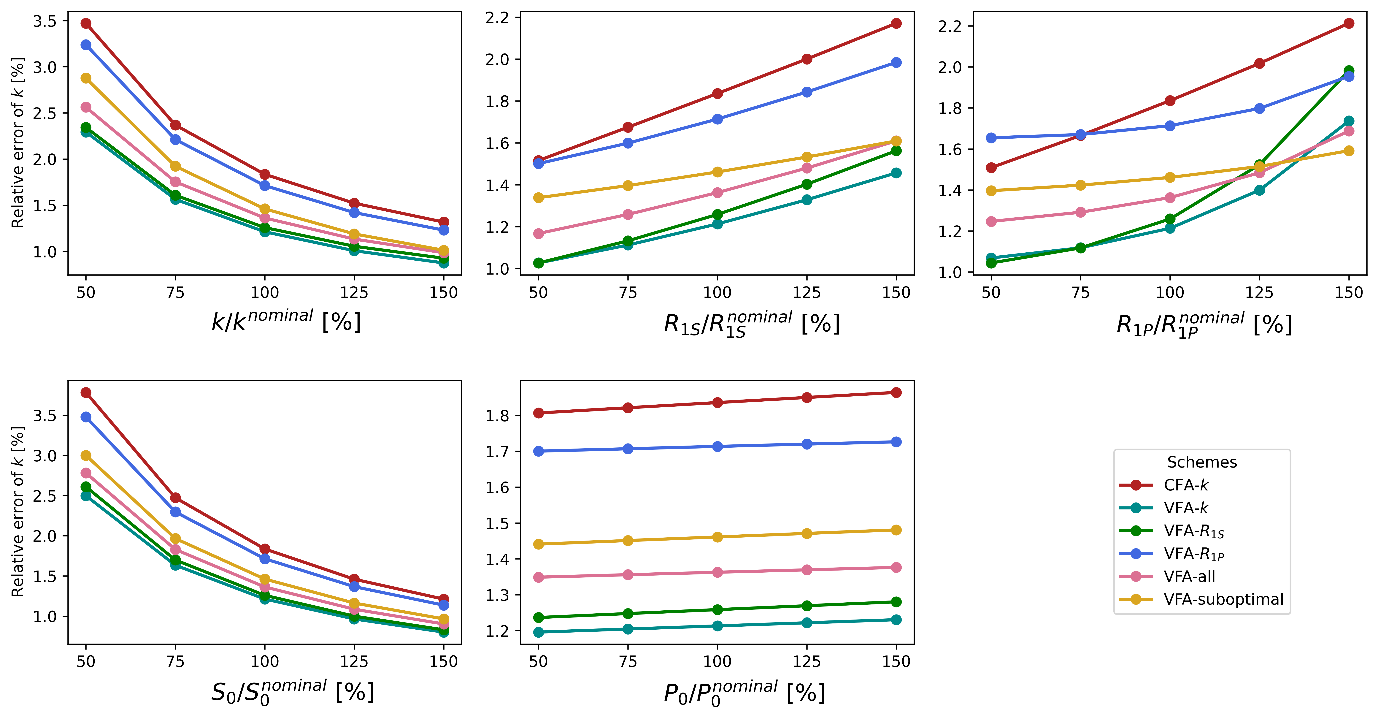


**Figure S5:** The relative error of the fitted *k* for all excitation schemes. Each of the points are averages based on 1000 realizations of simulated data. The horizontal axis indicates a selection of simulated ground truth parameters deviating from the common nominal parameter values used for flip angle optimization. Data were evaluated for five simulated ground truth parameters ranging from 50% to 150% to verify that the measurements conducted with the optimized flip angle schemes are suited for parameter estimation, also when there is substantial mismatch between all actual parameters and the expected parameter values.


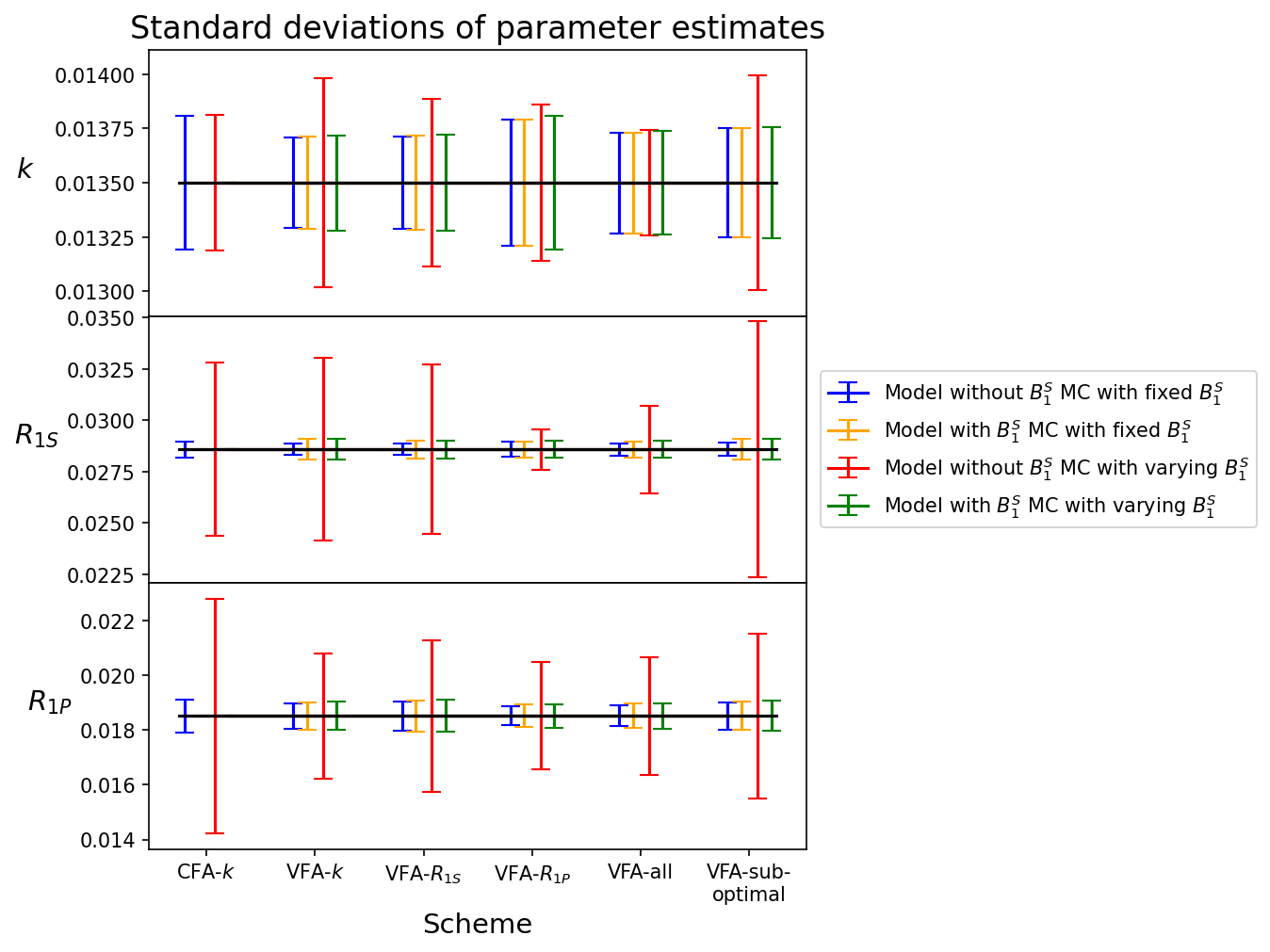


**Figure S6:** Standard deviation of rate parameters generated through MC simulations utilizing the schemes specified in Table 1. The blue and yellow bars display standard deviations obtained by fixing $B_{1}^{S}=1$ in the MC-generated data and respectively either excluding or including $B_{1}^{S}$-fitting in the data analysis. The red and green bars display standard deviations obtained by sampling $B_{1}^{S}$ at random from a normal distribution and respectively either excluding or including $B_{1}^{S}$-fitting in the data analysis. Introducing variance in $B_{1}^{S}$ and not subsequently fitting it, results in large variance of the other fitted parameters due to the correlation between parameter estimates.


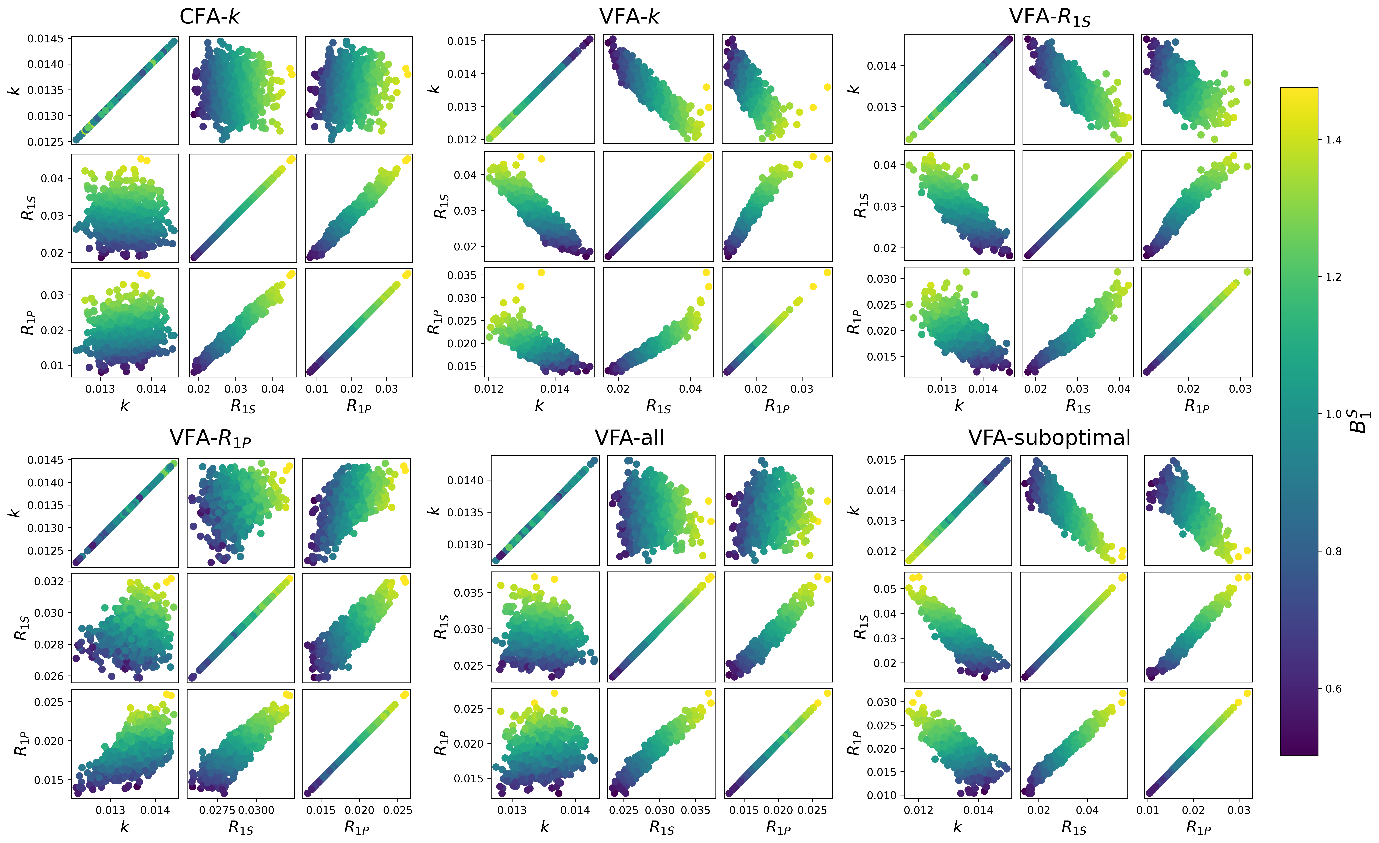


**Figure S7:** Scatter plots of MC estimated model rate parameters plotted pairwise against each other utilizing the six optimized flip angle schemes and the model without the $B_{1}^{S}$parameter. These same parameter estimates were used to calculate the red error bars in Figure S2. The color-gradient indicates the size of fitted $B_{1}^{S}$. Outliers were removed based on the parameter $k$ being more than 5 standard deviations off.

1. Borga M, Investigating the agreement between methods of different precision (2022). arXiv preprint arXiv: 2212.11769 [↑](#footnote-ref-1)
